# Two-step care pathways for advanced MASLD in primary and hospital care: A multicenter study

**DOI:** 10.1097/HC9.0000000000000976

**Published:** 2026-06-12

**Authors:** Stan Driessen, Koen C. van Son, Tamara R. Riemersma, Emily Stramigioli, Gerlinde L.G. Haverkamp, Maaike J. Denters, Gabrielle Alblas, Eric T.T.L. Tjwa, Nordin M.J. Hanssen, Sara-Joan Pinto-Sietsma, Yasaman Vali, Henrike M. Hamer, Laurens A. van Kleef, Diana J. Leeming, Ida Lønsmann, Morten A. Karsdal, Manuel Castro Cabezas, R. Bart Takkenberg, Willem P. Brouwer, Henk J. Schers, Max Nieuwdorp, Joost P.H. Drenth, Maarten E. Tushuizen, Adriaan (Onno) G. Holleboom

**Affiliations:** 1 Department of Vascular Medicine, Amsterdam UMC, University of Amsterdam, Amsterdam, The Netherlands; 2 Amsterdam Gastroenterology Endocrinology Metabolism (AGEM) Institute, Amsterdam UMC, University of Amsterdam, Amsterdam, The Netherlands; 3 Department of Gastroenterology and Hepatology, Amsterdam UMC, University of Amsterdam, Amsterdam, The Netherlands; 4 Department of Internal Medicine, Zaans Medisch Centrum, Zaandam, The Netherlands; 5 Department of Gastroenterology and Hepatology, Zaans Medisch Centrum, Zaandam, The Netherlands; 6 Department of Gastroenterology and Hepatology, LUMC, Leiden University, Leiden, The Netherlands; 7 Department of Gastroenterology and Hepatology, Radboudumc, Radboud University, Nijmegen, The Netherlands; 8 Department of Epidemiology and Data Science, Amsterdam UMC, University of Amsterdam, Amsterdam, The Netherlands; 9 Department of Laboratory Medicine, Laboratory Specialized Diagnostics & Research, Amsterdam UMC, University of Amsterdam, Amsterdam, The Netherlands; 10 Department of Gastroenterology and Hepatology, Erasmus MC, Erasmus University Rotterdam, Rotterdam, The Netherlands; 11 Nordic Bioscience A/S, Biomarkers & Research, Herlev, Denmark; 12 Department of Internal Medicine, Franciscus Gasthuis and Vlietland, Rotterdam, The Netherlands; 13 Department of Internal Medicine and Endocrinology, Erasmus MC, Erasmus University Rotterdam, Rotterdam, The Netherlands; 14 Julius Center for Health Sciences and Primary Care, UMC Utrecht, Utrecht University, Utrecht, The Netherlands; 15 Department of Primary and Community Care, Radboudumc, Radboud University, Nijmegen, The Netherlands

**Keywords:** biomarkers, clinical pathways, early diagnosis, elastography, metabolic dysfunction–associated steatohepatitis

## Abstract

**Background:**

Early diagnosis of metabolic dysfunction–associated steatotic liver disease (MASLD) fibrosis may prevent liver-related morbidity and mortality. We investigated the effectiveness of multiple 2-step approaches for detecting at-risk advanced MASLD fibrosis, defined as a hierarchical clinical score comprising liver histology and VCTE depending on availability, in primary and hospital outpatient care.

**Methods:**

Patients at risk for MASLD were recruited from general practices and regional and tertiary internal medicine clinics, after excluding other liver diseases. Simultaneous measurement of non-invasive tests (NITs), including fibrosis-4 score (FIB4), metabolic dysfunction–associated fibrosis-5 score (MAF5), NAFLD fibrosis score (NFS), enhanced liver fibrosis (ELF) test, procollagen type III N-terminal propeptide (PRO-C3), procollagen type VI N-terminal propeptide (PRO-C6), and vibration-controlled transient elastography (VCTE), allowed testing of 2-step referral pathways. Referral patterns were compared with regular care between 2016 and 2024 using a predefined reference standard.

**Results:**

Among 595 participants, 9.4% were at-risk of advanced MASLD fibrosis. A care pathway consisting of FIB4 (≥1.30) followed by VCTE yielded the greatest improvement, increasing the correct referral rate 5.0-fold (95% CI: 2.4–10.1) compared with regular care. However, care pathways incorporating MAF5 (≥1.0) or NFS (≥−1.455) followed by VCTE demonstrated markedly higher sensitivity at comparable increases in detection (both >4.5-fold). Among other second-step NITs, PRO-C3 outperformed ELF, PRO-C6, and ADAPT, resulting in a 2.2-fold (95% CI: 1.1–4.4) increase in correct referrals when used after FIB4 (≥1.30).

**Conclusion:**

Two-step care pathways substantially improve the detection of at-risk advanced MASLD fibrosis across care settings. Pathways using FIB4, MAF5, or NFS followed by VCTE offer the best performance overall, while PRO-C3 is the most effective alternative blood-based second-step test.

## Graphical abstract

**Figure:**
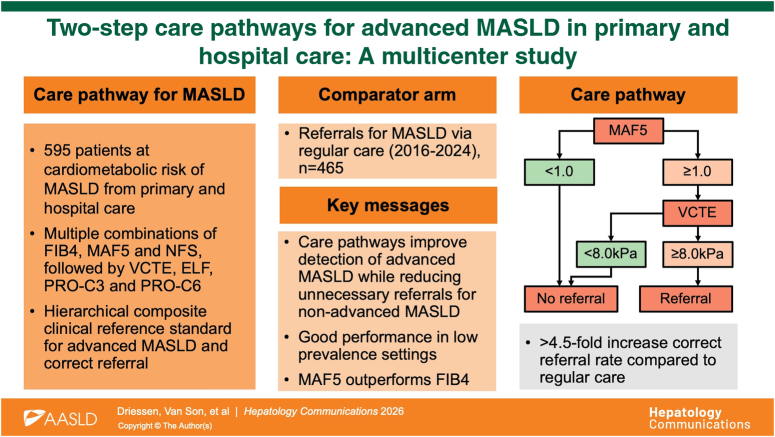

## INTRODUCTION

In concert with the increase in obesity and type 2 diabetes mellitus (T2DM), metabolic dysfunction–associated steatotic liver disease (MASLD) has become the most prevalent chronic liver disease worldwide, affecting approximately one-third of the global population.^1^ MASLD may progress to advanced fibrosis, cirrhosis, and hepatocellular carcinoma, but frequently remains unnoticed by both patients and physicians since specific symptoms are often absent until cirrhosis and its complications develop.^2^ Early diagnosis of fibrosis and subsequent appropriate management have the potential to prevent such progression to cirrhosis.^3^ Early identification requires vigilance and calls for dedicated care pathways to identify patients with advanced MASLD in at-risk populations.

Guidelines advocate the use of non-invasive tests (NITs) that allow for the detection of advanced fibrosis, including cirrhosis in MASLD.^3^,^4^ The fibrosis-4 score (FIB4), a composite score requiring age, aspartate aminotransaminase (AST), alanine aminotransaminase (ALT), and platelets, is the best-established non-patented blood-based tool to screen for MASLD fibrosis. The metabolic dysfunction–associated fibrosis-5 score (MAF5) is a recently developed alternative to FIB4, tailored for low-prevalence populations and requires waist circumference, body mass index (BMI), AST, platelets, and the presence of T2DM.^5^ In addition, vibration-controlled transient elastography (VCTE) by FibroScan^6^ and blood-based biomarkers^7^,^8^,^9^ are used for detecting fibrosis in the context of MASLD.

The optimal screening strategy for the detection of MASLD fibrosis in low-prevalence populations is unclear^3^ and depends on the a priori probability of advanced fibrosis, which in turn is related to the line of care. Sequential screening strategies are emerging as advantageous for the identification of high-risk individuals. They bank on the presence of a broadly applicable entry screening test, followed by a more specific and resource-intensive test to confirm the presence of fibrosis. Earlier studies have evaluated sequential screening strategies for MASLD, primarily relying upon FIB4 as the entry test.^10^,^11^,^12^,^13^,^14^,^15^ In addition, no direct comparison of care pathway implementation between primary care and the hospital outpatient clinic settings has been reported yet, and direct comparison to regular care is lacking. In addition, the reluctance of primary care providers and hospital specialists outside hepatology to screen for MASLD fibrosis, largely driven by the high volume of at-risk patients, highlights the need for clear clinical care pathways transcending traditional specialty boundaries. To address these matters, we designed a strategy to improve case finding of patients at cardiometabolic risk of MASLD while reducing the number of referrals to hepatology outpatient clinics for patients with absent or mild fibrosis. We investigated the effectiveness of 2-step approaches in a primary and hospital outpatient clinic care setting in the Netherlands, and compared this to regular care.

## METHODS

### Study design and participant selection

The study had 2 arms: a prospective care pathway study and a retrospective evaluation of regular care referrals. The prospective arm involved 51 general practitioners (GPs), 5 regional hospitals, and 2 academic hospitals in the Netherlands and ran from November 2022 until June 2024. The study followed the Declaration of Helsinki and Istanbul, was approved by the Amsterdam UMC ethics committee, and was registered in ClinicalTrials.gov (NCT05712603).

GPs and internal medicine specialists recruited patients ≥18 years based on regular referral behavior and additional cardiometabolic and hepatic criteria in accordance with the screening recommendations outlines in the European Association for the Study of the Liver (EASL) MASLD guideline,^3^ either: T2DM, metabolic syndrome,^16^ obesity, elevated ALT or hepatic steatosis on conventional ultrasound, in the absence of other causes of chronic liver disease and excessive alcohol use (≥21 units of alcohol/week in men and ≥14 units/week in women). These indications were treated as suggestions rather than strict inclusion or exclusion criteria to lower the threshold for referral. All participants gave written informed consent.

### Study visits and procedures

Participants attended a single study visit at Amsterdam UMC, Radboudumc, or Zaans Medisch Centrum (ZMC) after a ≥ 4-hour fast. The following data were collected: controlled attenuation parameter (CAP) and liver stiffness measurement (LSM) by VCTE using FibroScan, anthropometric measurements, measurements of NITs including FIB4, MAF5, and NAFLD fibrosis score (NFS), and self-reported weekly alcohol consumption. VCTE was performed by trained researchers using either the M-probe or XL-probe according to standard recommendations to ensure accurate measurements. LSM readings with at least 10 consecutive measurements and an interquartile range <30% were regarded as reliable. Additional blood samples were collected and stored in a designated biobank.

**FIGURE 1:**
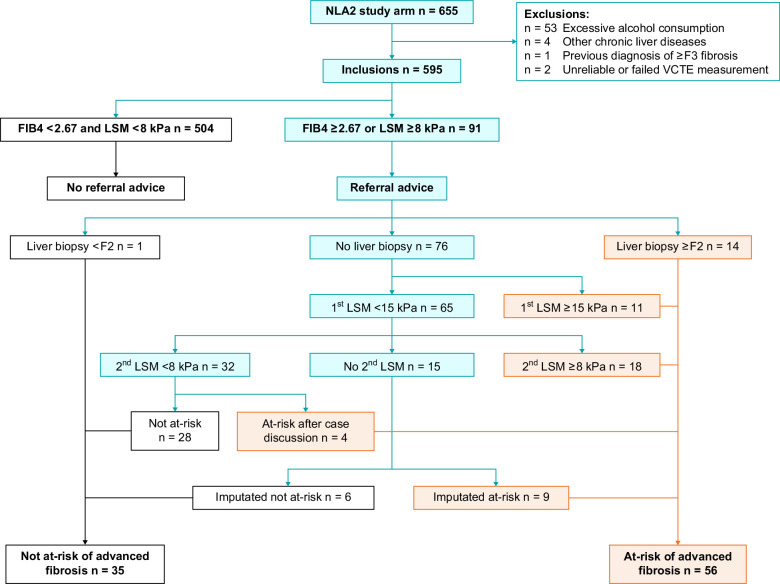
Flowchart and risk evaluation of NLA2 study arm participants. FIB4 ≥2.67 or LSM ≥8.0 kPa indicated a recommendation for referral to hepatology. At-risk or at low-risk of advanced fibrosis was determined using a predefined hierarchical clinical reference standard comprising liver histology, imaging parameters, and VCTE, depending on availability. Abbreviations: FIB4, fibrosis-4 index; LSM, liver stiffness measurement; VCTE, vibration-controlled transient elastography.

FIB4 ≥2.67 or LSM ≥8.0 kPa prompted a recommendation for referral to a hepatology clinic. Since all NITs were measured during a single visit, the lower rule-out FIB4 cutoff of 1.30, typically used as a screening threshold, was not applied. Instead, the rule-in threshold of 2.67 was used to reflect the referral threshold in a 2-step algorithm as recommended by the EASL.^3^ Participants with both FIB4 <2.67 and LSM <8.0 kPa were considered at low-risk of advanced (≥F3) fibrosis and remained under the care of their attending physician (Figure 1). Enhanced liver fibrosis (ELF) test was determined in serum using the Atellica IM analyzer (Siemens Healthineers) at Amsterdam UMC according to the manufacturer’s instructions.^17^,^18^ Procollagen type III N-terminal propeptide (PRO-C3) and procollagen type VI N-terminal propeptide (PRO-C6) were determined in serum using an enzyme-linked immunosorbent assay at the Nordic Bioscience laboratory, Herlev, Denmark.^8^ ELF, PRO-C3, and PRO-C6 were determined in bulk and were therefore not included in the study referral algorithm.

### Regular care comparator arm

All regular care referrals to 3 tertiary hepatology clinics (Amsterdam UMC, Radboudumc, and Leiden University Medical Center (LUMC)) from January 2016 to July 2024 were evaluated to assess the proportion of referred patients at-risk of advanced fibrosis. Referrals related to MASLD were included. The rigorous selection criteria are provided in Supplemental Figure S1 and the Supplemental Methods, http://links.lww.com/HC9/C359. Cases with a single LSM ≥8.0 kPa were excluded.

### Clinical reference standard

In both study arms, patients were classified as either at-risk or at low-risk of advanced MASLD fibrosis, using a predefined hierarchical clinical reference standard comprising liver histology, ultrasound, and VCTE, depending on availability. The endpoint was defined as “at-risk of advanced MASLD fibrosis” rather than strictly histological ≥F3 fibrosis, as it was designed to reflect clinically meaningful risk in routine specialist care rather than histology alone. The criteria were: histological fibrosis ≥F2, signs of cirrhosis on ultrasound, 2 or more LSMs ≥8.0 kPa with an interval <6 months, or a single LSM ≥15 kPa. Those referred without meeting these criteria were considered at low risk. Cases with a single LSM ≥8.0 kPa were imputed (see “Statistical analyses” section) in the active arm and excluded in the comparative arm. Records were reviewed by 2 researchers, with consensus sought in a multidisciplinary team when needed. Participants with large discordance in LSMs combined with significant weight loss were re-evaluated. The criteria are presented in Figure 1 and detailed in Supplemental Figure S2 and Supplemental Methods, http://links.lww.com/HC9/C359.

### Statistical analyses

Statistical analyses were conducted in R (version 4.3.2), with significance at *p* <0.05. Spearman’s Rank correlations between NITs were calculated, and agreement between NITs was determined using predefined cutoffs accompanied by Cohen’s kappa coefficients.^6^,^18^,^19^,^20^ As PRO-C6 lacked a predefined cutoff for MASLD fibrosis, a threshold of 12.0 was used as it yielded a comparable proportion of at-risk classifications as PRO-C3. Univariate and multivariate regression analyses were conducted to assess the association of cardiometabolic risk factors with at-risk advanced fibrosis using Firth’s penalized logistic regression. The performance of multiple 2-step care pathway algorithms for the detection of at-risk advanced fibrosis was analyzed, and risk ratios (RR) were used to compare detection rates of care pathway algorithms with regular care. In addition, the use of FibroScan-AST score (FAST) and ADAPT as alternatives to VCTE and PRO-C3, respectively, was evaluated.^21^,^22^ Missing clinical reference standard data existed only in patients who had an indication to be referred to a hepatology clinic (ie, FIB4 ≥2.67 or LSM ≥8.0 kPa) but were not referred. Therefore, missing data were imputed using MICE in RStudio with predictive mean matching (PMM) using all parameters to avoid selection bias. Trace plots and boxplots comparing the observed versus missing data were employed to assess the validity and robustness of imputation.

## RESULTS

### Participants characteristics

A total of 655 patients were included in the NLA2 care pathway study; 270 (41.2%) from primary care, 156 (23.8%) from secondary care, and 229 (35.0%) from tertiary care. In all, 60 (9.2%) patients were excluded from analyses (53 due to excessive alcohol intake), leaving 595 participants to be included in the analyses (Figure 1). Among the participants, 274 (46.4%) patients were women, and the median age was 60.0 (IQR: 51.0–68.0) years (Table 1). Median BMI was 30.1 (IQR: 26.9–33.6) kg/m^2^, and 307 (51.4%) had a BMI ≥30 kg/m^2^. A total of 293 (49.1%) participants had T2DM with a median HbA1c of 58 (IQR: 51–67) mmol/mol (Supplemental Table S2, http://links.lww.com/HC9/C359). In total, 438 (73.9%) participants had CAP ≥248 dB/m, suggesting ≥S1 steatosis (Table 2). In 91 (15.3%) cases, treating physicians were advised to refer participants to a hepatology clinic per study protocol (3 based on FIB4, 82 on LSM, and 6 on both). There were no significant differences in NITs in participants from secondary and tertiary care, so these participants were clustered and are further referred to as hospital outpatient clinic care (Supplemental Table S1 and S3, http://links.lww.com/HC9/C359). Descriptive statistics of NITs stratified for the presence of T2DM are presented in Supplemental Table S4, http://links.lww.com/HC9/C359.

**TABLE 1:** Participants characteristics

	Entire study population
n	595
Age, years	60 (51–68)
Sex, n women (%)	274 (46.5)
Healthcare line, n (%)	
Primary care	242 (40.7)
Secondary care	164 (27.6)
Tertiary care	189 (31.8)
Weight, kg	91.6 (±18.5)
BMI, kg/m^2^	30.1 (26.9–33.6)
BMI categories, n (%)	
<25.0 kg/m^2^	64 (10.8)
25.0–30.0 kg/m^2^	226 (38.0)
30.0–35.0 kg/m^2^	197 (33.1)
≥35.0 kg/m^2^	108 (18.2)
Waist circumference, cm	
Women	106.6 (±13.2)
Men	108.9 (±13.5)
T2DM, n (%)	293 (49.2)
Hypertension, n (%)	403 (67.7)
Dyslipidemia, n (%)	425 (71.4)
(History of) CVD, n (%)	160 (26.9)
Metabolic syndrome, n (%)	432 (79.6)
Laboratory measurements	
Platelets, ×10^9^	264.3 (±70.1)
AST, U/L	25 (21–32)
ALT, U/L	28 (21–41)
γGT, U/L	30 (20–46)
ALP, U/L	79 (65–95)
Albumin, g/L	40 (38–42)
Fasting glucose, mmol/L	5.9 (5.2–7.4)
HbA1c, mmol/mol	45 (39–58)
Total cholesterol, mmol/L	4.63 (±1.32)
LDL, mmol/L	2.56 (±1.15)
HDL, mmol/L	
Women	1.38 (1.15–1.61)
Men	1.12 (0.95–1.34)
Triglycerides, mmol/L	1.34 (0.93–2.10)

Abbreviations: ALP, alkaline phosphatase; ALT, alanine aminotransferase; AST, aspartate aminotransferase; BMI, body mass index; CVD, cardiovascular disease; HbA1c, glycated hemoglobin; HDL, high-density lipoprotein; LDL, low-density lipoprotein; T2DM, type 2 diabetes mellitus; γGT, gamma-glutamyl transpeptidase.

**TABLE 2:** Descriptive statistics of NITs

	Entire study population
n	595
Steatosis NITs	
CAP, dB/m	294 (243–337)
CAP categories, n (%)	
<248 dB/m	155 (26.1)
248–260 dB/m	31 (5.2)
260–290 dB/m	92 (15.5)
≥290 dB/m	315 (53.1)
Fibrosis NITs	
FIB4	1.11 (0.82–1.46)
FIB4 categories, n (%)	
<1.30	359 (64.2)
1.30–2.67	191 (34.2)
≥2.67	9 (1.6)
MAF5	1.6 (0.2–2.9)
MAF5 categories, n (%)	
<0	114 (20.5)
0–1	102 (18.3)
≥1	341 (61.2)
NFS	−1.22 (±1.35)
NFS categories, n (%)	
<−1.455	231 (41.6)
−1.455–0.67	282 (50.8)
≥0.67	42 (7.6)
LSM, kPa	5.3 (4.2–6.7)
LSM categories, n (%)	
<8.0 kPa	507 (85.2)
8.0–15.0 kPa	71 (11.9)
≥15.0 kPa	17 (2.9)
ELF	9.2 (8.6–9.7)
ELF categories, n (%)	
<7.7	21 (3.7)
7.7–9.8	437 (76.0)
≥9.8	117 (20.3)
PRO-C3	12.0 (9.7–15.0)
PRO-C3 categories, n (%)	
<15.6	412 (73.0)
≥15.6	152 (27.0)
PRO-C6	9.6 (8.1–12.1)
PRO-C6 categories, n (%)	
<12.0	426 (74.3)
≥12.0	147 (25.7)

Abbreviations: CAP, controlled attenuation parameter; ELF, enhanced fibrosis test; FIB4, fibrosis-4 index; LSM, liver stiffness measurement; MAF5, metabolic dysfunction–associated fibrosis-5 score; NFS, NAFLD fibrosis score; NIT, non-invasive test; PRO-C3, procollagen type III N-terminal propeptide; PRO-C6, procollagen type VI N-terminal propeptide.

Based on the clinical reference standard, 56 participants were at-risk of advanced fibrosis (9.4% of the total cohort) (Figure 1). The distribution of criteria contributing to at-risk classification is presented in Supplemental Table S5, http://links.lww.com/HC9/C359. Two participants were classified as false-negative after additional medical record review revealed liver biopsy findings of ≥F2 fibrosis. Diagnostic accuracy of individual NITs for at-risk advanced fibrosis and correlations between NITs can be found in Supplemental Tables S6–S12, http://links.lww.com/HC9/C359.

### Regular care comparator arm

Between January 2016 and July 2024, a total of 4540 patients were referred to a hepatology clinic of the included referral centers, of whom 456 (10.0%) were referred for MASLD and thus comprise the regular care comparator arm. Among these, 65 (14.0%) patients met the criteria for at-risk advanced fibrosis as outlined above (Supplemental Table S13, http://links.lww.com/HC9/C359). As such, regular care had a correct referral rate of 14.0% and an incorrect referral rate of 86%. The correct referral rate was 12.8% for patients referred from primary care compared with 15.0% for patients referred for hospital outpatient care. The correct referral rate was 31.4% for patients with T2DM compared with 8.7% for patients without T2DM. The distribution of criteria contributing to at-risk classification is presented in Supplemental Table S5, http://links.lww.com/HC9/C359.

### Predictors of at-risk MASLD fibrosis

Multivariate regression analyses incorporating criteria of the metabolic syndrome identified obesity (BMI ≥30.0 kg/m^2^) as the strongest predictor of at-risk MASLD fibrosis with an OR of 7.7 (95% CI: 3.6–19.1, *p*<0.001) (Table 3). This was followed by T2DM, which had an OR of 2.3 (95% CI: 1.2–4.3, *p*=0.009). In univariate analyses, metabolic syndrome itself had an OR of 1.5 (95% CI: 0.74–3.6, *p*=0.309), while the number of cardiometabolic syndrome criteria showed a significant association with an OR of 1.5 (95% CI: 1.2–1.9, *p*<0.001). In addition, the mean number of cardiometabolic risk factors was higher among those with at-risk advanced fibrosis compared with those without [3.4 (±1.3) vs. 3.0 (±1.3), *p*=0.043]. Obesity remained the main predictor when stratified for the lines of care (Supplemental Table S14, http://links.lww.com/HC9/C359).

**TABLE 3:** Predictors of at-risk advanced MASLD fibrosis

		Univariate regression		Multivariate regression	
	n (%)	OR	*p*	OR	*p*
T2DM	293 (49.2)	2.1 (1.2–3.8)	0.008	2.3 (1.2–4.3)	0.009
Hypertension	403 (67.7)	1.5 (0.5–5.5)	0.483	1.4 (0.5–6.3)	0.573
Dyslipidaemia	435 (71.4)	0.7 (0.4–1.4)	0.336	0.7 (0.4–1.3)	0.208
Obesity	305 (51.3)	7.3 (3.5–17.4)	<0.001	7.7 (3.6–19.1)	<0.001
Metabolic syndrome	432 (79.6)	1.5 (0.7–3.4)	0.309	—	—

Abbreviations: MASLD, metabolic dysfunction–associated steatotic liver disease; OR, odds ratio; T2DM, type 2 diabetes mellitus.

### Two-step care pathways using VCTE

**FIGURE 2:**
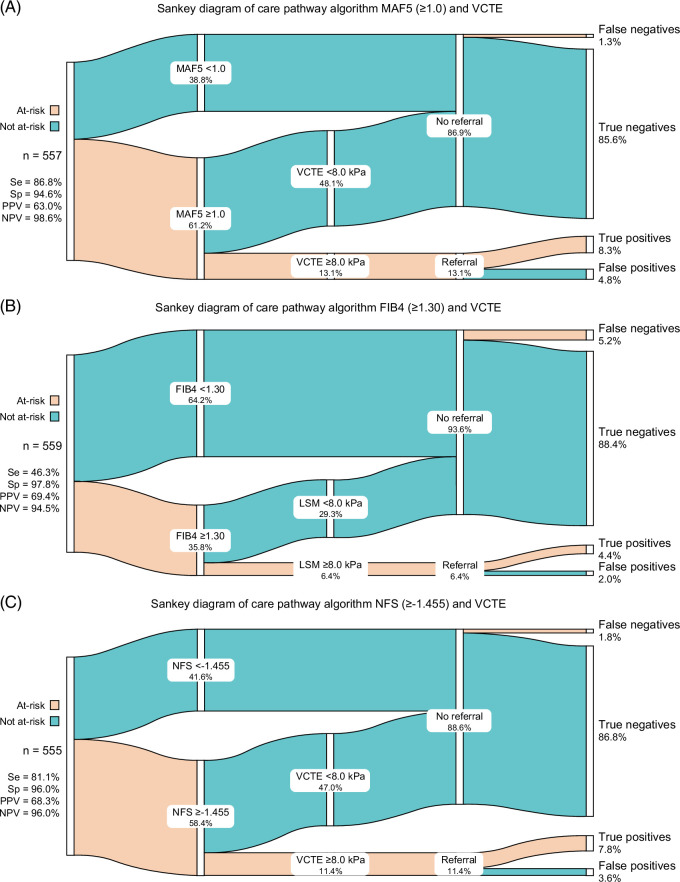
Sankey diagrams of different care pathway algorithms: MAF5 (≥1.0) (A), FIB4 (≥1.30) (B), and NFS (≥−1.455) (C), followed by VCTE (≥8.0 kPa). This figure presents Sankey diagrams illustrating patient flow through different diagnostic care pathways for MASLD-related fibrosis. Abbreviations: FIB4, fibrosis-4 index; MAF5, metabolic dysfunction–associated fibrosis-5 score; MASLD, metabolic dysfunction–associated steatotic liver disease; NFS, NAFLD fibrosis score; VCTE, vibration-controlled transient elastography.

Various multiple test care pathway scenarios were investigated, starting with a first-step NIT, that is, FIB4, MAF5, or NFS, followed by a second-step NIT, that is, VCTE, ELF, PRO-C3, PRO-C6, FAST, or ADAPT. In a care pathway consisting of FIB4 (≥1.30) and VCTE, 35.8% would require a VCTE, and 6.4% would be referred to hepatology, 69.4% of whom would be referred correctly (Figure 2 and Table 4). This would increase the correct referral rate 5.0-fold (95% CI: 2.4–10.1) compared with regular care. Simultaneously, the percentage of unnecessary referrals would decrease from 86.0% to 30.6%. This approach boosts a sensitivity of 0.46 (95% CI: 0.33–0.60) and a specificity of 0.98 (95% CI: 0.97–0.99).

**TABLE 4:** Diagnostic accuracy and performance of 2-step care pathways using VCTE

	VCTE needed, %	Referral to hepatology, %	PPV, % (95% CI)	NPV, % (95% CI)	Sensitivity, % (95% CI)	Specificity, % (95% CI)	Correct referral rate, %	Improved correct referral rate, RR (95% CI)	NNS for correct referral, whole care path, n	NNS for correct referral, after VCTE, n
FIB4/VCTE if FIB4 ≥1.30	35.8	6.4	69.4 (54.4–84.5)	94.5 (92.5–96.4)	46.3 (33.0–59.6)	97.8 (96.5–99.1)	69.4	5.0 (2.4–10.1)	22.4	8.0
MAF5/VCTE if MAF5 ≥1.0	61.2	13.1	63.0 (51.9–74.1)	98.6 (97.5–99.6)	86.8 (77.7–95.9)	94.6 (92.7–96.6)	63.0	4.5 (2.2–9.0)	12.1	7.4
NFS/VCTE if NFS ≥−1.455	58.4	11.4	68.3 (56.8–79.7)	98.0 (96.7–99.2)	81.1 (70.6–91.7)	96.0 (94.3–97.7)	68.3	4.9 (2.4–9.9)	12.9	7.5

Abbreviations: CI, confidence interval; FIB4, fibrosis-4 score; MAF5, metabolic dysfunction–associated fibrosis-5 score; NFS, NAFLD fibrosis score; NNS, number-needed-to-screen; NPV, negative predictive value; PPV, positive predictive value; RR, risk ratio; VCTE, vibration-controlled transient elastography.

A care pathway consisting of MAF5 (≥1.0) followed by VCTE would require more VCTEs (61.2%), and more patients would be referred to hepatology (14.2%). The correct referral rate would increase 4.5-fold (95% CI: 2.2–9.0) compared with regular care, and the percentage of unnecessary referrals would decrease to 37.0%. Sensitivity is markedly higher than that of a care pathway consisting of FIB4 (≥1.30) and VCTE at 0.87 (95% CI: 0.78–0.96), and specificity is comparable at 0.95 (95% CI: 0.93–0.97) (Figure 2A and Table 4).

A care pathway consisting of NFS (≥−1.455) and VCTE boosts similar results to one using MAF5 (≥1.0) and VCTE and would increase the correct referral rate 4.9-fold (95% CI: 2.4–9.9) with a sensitivity of 0.81 (95% CI: 0.71–0.92) (Figure 2C and Table 4). The use of FAST as the second-step test did not outperform VCTE with improved correct referral rates ranging from 2.2 (95% CI: 1.1–4.5) to 3.2 (95% CI: 1.6–6.3) when using MAF5 (≥1.0) and NFS (≥−1.455) as the first-step test, respectively (Supplemental Table S15, http://links.lww.com/HC9/C359).

### Stratifying for lines of care

In the primary care setting, a care pathway consisting of FIB4 (≥1.30) followed by VCTE would require VCTE in 38.8% of patients, and 4.7% would be referred to hepatology. The correct referral rate would increase 5.7-fold (95% CI: 2.7–11.8), and the number of unnecessary referrals would decrease from 87.2% to 27.3%. This approach yields a sensitivity of 0.53 (95% CI: 0.28–0.79) and a specificity of 0.99 (95% CI: 0.97–1.00) (Supplemental Table S16, http://links.lww.com/HC9/C359). A care pathway consisting of MAF5 (≥1.0) followed by VCTE would again require more VCTEs (50.4%) and would refer more patients to hepatology (7.3%) compared with a care pathway with FIB4 (≥1.30). The correct referral rate would increase 6.0-fold (95% CI: 2.8–12.6) compared with regular care. Sensitivity lies at 0.87 (95% CI: 0.70–1.00) and specificity at 0.98 (95% CI: 0.96–1.00) (Supplemental Table S17, http://links.lww.com/HC9/C359). A care pathway with NFS (≥−1.455) requires slightly more VCTEs (54.6%) compared with a care pathway with MAF5 (≥1.0) and boosts a comparable fold-increase in the correct referral rate, though at slightly lower sensitivity [0.80 (95% CI: 0.60–1.00)] (Supplemental Table S16, http://links.lww.com/HC9/C359).

A care pathway consisting of FIB4 (≥1.30) and VCTE would increase the correct referral rate 4.5-fold (95% CI: 2.3–9.0) compared with regular care when employed in the hospital outpatient care setting and would yield a sensitivity of 0.44 (95% CI: 0.28–0.59) (Supplemental Table S16, http://links.lww.com/HC9/C359). By comparison, a care pathway consisting of MAF5 (≥1.0) and VCTE would increase the correct referral rate 3.9-fold (95% CI: 2.0–7.7) and have a sensitivity of 0.92 (95% CI: 0.84–1.00) (Supplemental Table S16, http://links.lww.com/HC9/C359). Finally, employing a care pathway consisting of NFS (≥−1.445) and VCTE would increase the correct referral rate 4.4-fold (95% CI: 2.2–8.7) compared with regular care (Supplemental Table 16, http://links.lww.com/HC9/C359). Overall, the fold-increase in correct referrals is higher in the primary care setting compared with the hospital outpatient setting.

### Stratifying for T2DM

The employment of 2-tiered care pathways with VCTE as the second-tier test among patients with T2DM would increase the correct referral rate 2.0-fold (range: 2.0–2.1) (Supplemental Table S17, http://links.lww.com/HC9/C359). Care pathways using MAF5 (≥1.0) or NFS (≥−1.455) as the first-tier test would require a subsequent VCTE in a majority of patients (84.7% and 79.2%, respectively). A care pathway with FIB4 (≥1.30) would require less VCTEs but boosts lower sensitivity at 0.49 (95% CI: 0.32–0.65) compared with 0.91 (95% CI: 0.82–1.00) and 0.94 (95% CI: 0.87–1.00), respectively (Supplemental Table S17, http://links.lww.com/HC9/C359).

In patients without T2DM, a care pathway consisting of FIB4 (≥1.30) and VCTE would increase the correct referral rate 9.2-fold (95% CI: 3.9–21.5) and have a sensitivity of 0.42 (95% CI: 0.20–0.64). In comparison, a care pathway consisting of MAF5 (≥1.0) and VCTE would increase the correct referral rate 7.2-fold (95% CI: 3.2–16.1) and have a sensitivity of 0.79 (95% CI: 0.61–0.97) and one consisting of NFS (≥−1.455) would increase the correct referral rate 9.6-fold (95% CI: 4.0–22.9) and have a sensitivity of 0.56 (95% CI: 0.33–0.79) (Supplemental table 17, http://links.lww.com/HC9/C359).

### Two-step care pathways with other second-step NITs than VCTE

**FIGURE 3:**
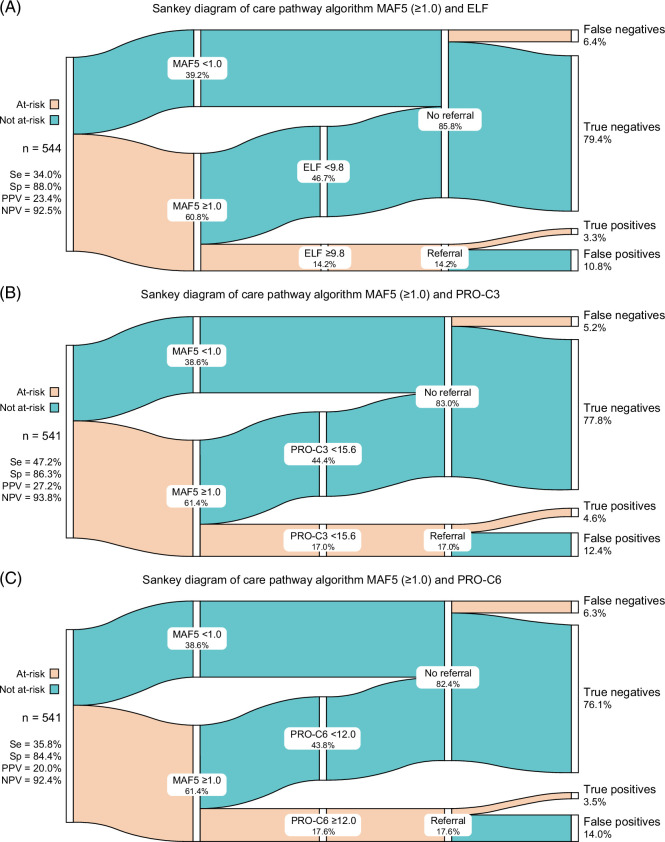
Sankey diagrams of different care pathway algorithms: MAF5 (≥1.0) followed by either ELF (A), PRO-C3 (B), and PRO-C6 (C). This figure presents 3 Sankey diagrams illustrating patient flow through different diagnostic care pathways for MASLD-related fibrosis. Abbreviations: ELF, enhanced liver fibrosis test; MAF5, metabolic dysfunction–associated fibrosis-5 score; MASLD, metabolic dysfunction–associated steatotic liver disease; PRO-C3, procollagen type III N-terminal propeptide; PRO-C6, procollagen type VI N-terminal propeptide.

In addition to care pathways employing VCTE as the second-tier test, we also conducted in silico evaluations of care pathways incorporating blood-based NITs, namely ELF, PRO-C3, PRO-C6, and ADAPT (Figure 3). A care pathway consisting of FIB4 (≥1.30) followed by PRO-C3 (≥15.6) would improve the correct referral rate 2.2-fold (95% CI: 1.1–4.4) compared with regular care (Supplemental Table S18, http://links.lww.com/HC9/C359). When performed in the primary care setting, this fold-improvement would increase to 3.1 (95% CI: 1.5–6.4) (Supplemental Table S19, http://links.lww.com/HC9/C359). Care pathways using ELF, PRO-C6, or ADAPT as the second-tier test do not increase correct referral rates compared with regular care (Supplemental Tables S20–S26, http://links.lww.com/HC9/C359).

## DISCUSSION

In this prospective multicenter care pathway study, we demonstrated that 2-step care pathways considerably improve the detection of at-risk MASLD fibrosis compared with regular care. Using FIB4, MAF5, or NFS as first-step NITs followed by VCTE increases the correct referral rate while reducing unnecessary referrals to hepatology. All 3 approaches lowered the number of required VCTEs, with FIB4 showing the greatest reduction, but at the cost of lower sensitivity and more false negatives compared with MAF5 or NFS. Overall, care pathways consisting of MAF5 or NFS followed by VCTE demonstrated the best performance by increasing the correct referral rate compared with regular care while maintaining high sensitivity. In the absence of VCTE, PRO-C3 outperforms ELF, PRO-C6, and ADAPT as the second-step test. Importantly, NIT selection should be tailored to the clinical setting and available resources: MAF5 requires waist measurement, NFS albumin, and VCTE necessitates easy access, trained personnel, and specialized equipment. In contrast, using blood-based biomarkers as a second-step test may enable reflex testing, allowing automatic follow-up after elevated first-step results.

We found that the diagnostic performance of each strategy strongly depends upon the care setting and the presence of T2DM. Notably, the increase in relative risk (RR) for detecting at-risk advanced fibrosis using care pathways was driven by the absence of T2DM. This reflects the low detection rate of MASLD in regular care. These findings shed light on the understudied performance of NITs in low-prevalence populations, a key research topic according to EASL,^3^ and support their targeted use in metabolically at-risk populations, regardless of T2DM presence. Furthermore, implementation of care pathways within primary care substantially increases the correct referral rate compared with regular care. While this approach increases the number of VCTEs required in primary care, it should also be noted that MASLD is a slowly progressive disease. Therefore, examinations would only need to be repeated at intervals of several years, which may mitigate the resource burden.

At present, FIB4 is widely recommended as a first-step test due to its ease of use, low cost, and large body of evidence.^3^,^4^,^23^ We demonstrated marked differences between FIB4 and MAF5; FIB4 doubles the number-needed-to-screen (NNS) compared with MAF5, but MAF5 requires 1.8-fold more second-step tests. Notably, pathways using MAF5 or NFS had a lower NNS per second-step test than FIB4, suggesting lower use of health care resources per detected case despite more false positives. While previous studies have used LSM as a reference standard, VCTE can vary by operator and over time.^24^,^25^ To improve accuracy, we required at least 2 consecutive VCTE measurements to classify at-risk advanced fibrosis. Over 30% of participants with an initial LSM ≥8.0 kPa had <8.0 kPa on repeat measurement in both study arms, supporting the added value of repeating VCTE in practice.

This study was designed to capture real-world referral behavior, focusing on the actual healthcare implications of different care pathways. Referred participants underwent standard clinical workup for MASLD at the hepatology outpatient clinic, rarely including liver biopsy or magnetic resonance elastography (MRE). Consequently, we reverted to a predefined hierarchical clinical reference standard to construct our primary endpoint. The reported predictive values, therefore, reflect how well these care pathways align with the current practices and standards in specialized liver care rather than with histological features of MASLD. This nuances the relatively poor performance of care pathways utilizing ELF, PRO-C3, PRO-C6, and ADAPT as a second-step test, given the poor agreement between these blood-based biomarkers and VCTE and the latter being part of the evaluation standard. Nonetheless, these parameters have limited performance in identifying patients with consecutively elevated VCTE. A healthcare system that relies on VCTE for hepatology care but lacks the capacity to perform VCTEs in other settings may benefit most from using PRO-C3 as a more scalable alternative, for example, in diagnostic laboratories, allowing the use of reflex testing algorithms. Although FAST and ADAPT were developed to enhance the diagnostic accuracy of VCTE and PRO-C3, respectively, their added benefit was not evident within the context of 2-step care pathways. This is most likely due to the fact that variables used in these scores, such as liver enzymes and platelet count, are already incorporated into the first-step NITs. The relatively modest correlation between different NITs likely reflects that they capture related but distinct biological processes, for example, mechanical stiffness measured by VCTE and matrix turnover and fibrogenesis reflected by circulation biomarkers, while also being influenced by different sources of technical, analytical, and biological variability. In addition, range restriction in low-prevalence populations, where most individuals cluster within the low-risk spectrum, may further attenuate correlation coefficients and limit agreement.

Our study has several strengths. Including both primary care and hospital outpatient care in multiple regions enabled the first rigorous comparison of care pathways across care settings. Comprehensive multicenter screening of regular care hepatology referrals provided a reliable estimate of additional benefits. While using VCTE in both the referral algorithm and the reference standard introduced incorporation bias, the reference standard aligns with current hepatology practice, thus not limiting clinical utility. All patients underwent VCTE and blood-based NITs during a single study visit, enabling NPV estimations of individual NITs. In current practice, physicians refer patients based on a variety of criteria. Even if future screening shifts toward cardiometabolic risk factors, a heterogeneous referral pattern, including referrals based solely on elevated liver enzymes, is likely to persist. Our recommendation to recruit patients using both cardiometabolic criteria and following previously routine referral patterns simulates this anticipated future scenario.

In terms of limitations, the reference standard’s requirement for multiple VCTE measurements excluded cases with only one LSM ≥8.0 kPa, which may have led to an underestimation of at-risk advanced fibrosis in the regular care arm. Furthermore, patients classified as low-risk per protocol did not receive a second VCTE, potentially underestimating false negatives since additional workup within these patients is rare.

Our findings highlight the significant impact of first-step NIT choice for the detection of at-risk advanced fibrosis and referral rates to specialized liver care. Of note, as obesity and T2DM prevalence increase in younger populations,^26^ the diagnostic accuracy of age-dependent FIB4 is expected to decline. While FIB4 is widely used, there remains room for improvement in non-invasive fibrosis assessment. Several newer fibrosis scores, such as LiverPRO and LiverRisk, have shown promising diagnostic potential; however, these tools are not open-source and have limited independent external validation.^27^,^28^ Future studies should evaluate their utility and reproducibility across diverse healthcare settings. Ultimately, long-term follow-up of care pathway studies may determine which diagnostic algorithm leads to the largest reduction in morbidity, mortality, and increase in quality-adjusted life years (QALYs), prerequisites for a bona fide cost-effectiveness analysis. The burgeoning obesity pandemic is estimated to add $4.3 trillion to global annual healthcare costs by 2035,^29^ with fibrotic MASLD contributing to these increasing numbers.^30^ Thus, care pathways will substantially influence healthcare costs, and cost-effectiveness analyses will be crucial in guiding national secondary prevention strategies. Several cost-effectiveness studies on fibrosis screening exist in the literature.^31^ These studies are modeled under the assumption that diagnosing MASLD fibrosis will reduce the rate of progression, which is likely but remains to be demonstrated.

In conclusion, our findings show that any 2-step care pathway tremendously improves the detection of at-risk advanced MASLD fibrosis compared to regular care. Simultaneously, more patients may be reassured that they do not have advanced fibrosis, thus halving the number of referrals of patients at low risk of disease to specialized liver care. A 2-step care pathway of MAF5 or NFS followed by VCTE has the best performance by increasing the correct referral rate compared with regular care while maintaining high sensitivity. Care pathways are effective in the low-prevalent settings of primary care and in people without T2DM, and the use of cardiometabolic risk factors may help identify those patients most at risk of severe MASLD.

## Supplementary Material
