# Supplementary material for: Two-step care pathways for advanced MASLD in primary and hospital care: A multicenter study
Source: Hepatol Commun. 2026 Jun 12;10(7):e0976. doi: 10.1097/HC9.0000000000000976 (PMC13263074; doi:10.1097/HC9.0000000000000976)
Supplement: Supplementary file 1 [file hc9-10-e0976-s001.docx]

**Supplementary material**

**Table of contents:**

[**Supplemental methods** 3](#_Toc222994832)

[**Supplemental table 2.** Participant characteristics stratified for the presence of T2DM. 6](#_Toc222994833)

[**Supplemental table 3.** Descriptive statistics non-invasive tests, stratified for the lines of care. 8](#_Toc222994834)

[**Supplemental table 4.** Descriptive statistics non-invasive tests, stratified for the presence of T2DM. 10](#_Toc222994835)

[**Supplemental table 6.** Diagnostic accuracy results and performance of individual NITs for at-risk advanced fibrosis. 13](#_Toc222994836)

[**Supplemental table 8.** Diagnostic accuracy results and performance of individual NITs for at-risk advanced fibrosis, hospital outpatient clinic care. 17](#_Toc222994837)

[**Supplemental table 9.** Diagnostic results and performance of individual NITs for at-risk advanced fibrosis, T2DM present. 19](#_Toc222994838)

[**Supplemental table 10.** Diagnostic results and performance of individual NITs for at-risk advanced fibrosis, T2DM absent. 21](#_Toc222994839)

[**Supplemental table 11.** Spearman’s correlation non-invasive tests. 23](#_Toc222994840)

[**Supplemental table 12.** Agreement VCTE (LSM), ELF, PRO-C3 and PRO-C6, determined by Cohen’s kappa. 24](#_Toc222994841)

[**Supplemental table 13.** Participant characteristics comparative arm. 25](#_Toc222994842)

[**Supplemental table 15.** Diagnostic results and performance two-tiered care pathways using FAST and ADAPT. 27](#_Toc222994843)

[**Supplemental table 16.** Diagnostic results and performance two-tiered care pathways using VCTE, stratified for the lines of care. 28](#_Toc222994844)

[**Supplemental table 17.** Diagnostic results and performance two-tiered care pathways using VCTE, stratified for the presence of T2DM. 29](#_Toc222994845)

[**Supplemental table 18.** Diagnostic results and performance two-tiered care pathways using PRO-C3. 30](#_Toc222994846)

[**Supplemental table 19.** Diagnostic results and performance two-tiered care pathways using PRO-C3, stratified for the lines of care. 31](#_Toc222994847)

[**Supplemental table 20.** Diagnostic results and performance two-tiered care pathways using PRO-C3, stratified for the presence of T2DM. 32](#_Toc222994848)

[**Supplemental table 21.** Diagnostic results and performance two-tiered care pathways using ELF. 33](#_Toc222994849)

[**Supplemental table 22.** Diagnostic results and performance two-tiered care pathways using ELF, stratified for the lines of care. 34](#_Toc222994850)

[**Supplemental table 23.** Diagnostic results and performance two-tiered care pathways using ELF, stratified for the presence of T2DM. 35](#_Toc222994851)

[**Supplemental table 24.** Diagnostic results and performance two-tiered care pathways using PRO-C6. 36](#_Toc222994852)

[**Supplemental table 25.** Diagnostic results and performance two-tiered care pathways using PRO-C6, stratified for the lines of care. 37](#_Toc222994853)

[**Supplemental table 26.** Diagnostic results and performance two-tiered care pathways using PRO-C6, stratified for the presence of T2DM. 38](#_Toc222994854)

[**Supplemental figure 1.** Flow chart exclusion criteria regular care arm. 39](#_Toc222994855)

[**Supplemental figure 2.** Flow chart clinical reference standard regular care arm. 40](#_Toc222994856)

# **Supplemental methods**

**Participating centers**

Participants were recruited from primary care setting in the regio of Amsterdam and Nijmegen and from internal medicine outpatient clinics from five regional hospitals (Amstelland ziekenhuis, Flevoziekenhuis, Onze Lieve Vrouwen Gasthuis (OLVG), Tergooi ziekenhuis and Zaans Medisch Centrum (ZMC)) and two academic hospitals (Amsterdam University Medical Centers (Amsterdam UMC) and Radboud University Medical Center (Radboudumc)). Regular care referrals to three tertiary hepatology clinics (Amsterdam UMC, Radboudumc and Leiden University Medical Center (LUMC) were included in the study.

**Regular care comparator arm**

All referrals to the tertiary hepatology clinics, i.e. from primary, secondary, and tertiary care, were evaluated. Those referrals with a reason related to MASLD - specifically, referrals explicitly mentioning MASLD, those with an established diagnosis of steatosis, or those with liver function tests indicating steatosis (elevated AST and/or ALT) in the absence of other chronic liver diseases - were included in the comparison analysis. Patients were excluded when they had a previous diagnosis of ≥F3 fibrosis, when they were referred by a gastroenterologist, when a VCTE was performed prior to the referral, or when the first VCTE was performed ≥3 months after initial visit at the outpatient clinic (Supplementary figure 1).

**Clinical reference standard**

When a liver biopsy was performed as part of the diagnostic work-up, those with histological fibrosis stage ≥F2 were classified as at-risk of advanced fibrosis. In absence of liver biopsy, those who underwent a conventional ultrasound showing signs of cirrhosis were regarded at-risk of advanced fibrosis. Since LSM can have variability (14), only participants with two or more LSM ≥8.0 kPa were classified to be at-risk of advanced fibrosis. Patients who were referred to the hepatology clinic and did not fulfill these criteria were classified as low-risk of advanced fibrosis. When patients had an initial LSM ≥8.0 kPa but lacked a second LSM, records were excluded from analyses, unless LSM was ≥15 kPa, indicative of cirrhosis. Records of referrals were evaluated by two different researchers (S.D., K.v.S.) and when necessary, consensus was reached in the study team consisting of the two researchers, one internist (A.H.) and two hepatologists (M.T., J.D.). In case of wide variation in the LSM readings and/or considerable weight loss between the first and second VCTE due to point-of-care effect of VCTE, the case was also discussed in the study team (Supplementary figure 2). Four participants were reclassified after case discussion in the active study arm and seven in the comparative arm. All achieved >10% body weight reduction between first and second LSM or had a third LSM >8.0 kPa. Medical records of participants deemed at low-risk of advanced MASLD-fibrosis were reviewed to assess whether advanced fibrosis or cirrhosis was overlooked; if this was the case, these cases were reclassified as at-risk of advanced MASLD-fibrosis.

| **Supplemental table 1.** Participant characteristics stratified for the lines of care. | | | | |
| --- | --- | --- | --- | --- |
|  |  | **Primary care** | **Hospital care** | **p-value** |
| n |  | 242 | 353 |  |
| Age, years (median (IQR)) |  | 63 (57-69) | 58 (49-66) | <0.001 |
| Sex, n women (%) |  | 123 (51.0) | 151 (43.4) | 0.078 |
| Weight, kg (mean (SD)) |  | 86.9 (17.1) | 94.8 (18.7) | <0.001 |
| BMI, kg/m^2^ (median (IQR)) |  | 28.5 (26.2-32.2) | 31.1 (28.0-34.6) | <0.001 |
| BMI categories, n (%) | <25.0 kg/m^2^ | 37 (15.3) | 27 (7.7) | <0.001 |
|  | 25.0-30.0 kg/m^2^ | 108 (44.6) | 118 (33.4) |  |
|  | 30.0-35.0 kg/m^2^ | 69 (28.5) | 128 (36.3) |  |
|  | ≥35.0 kg/m^2^ | 28 (11.6) | 80 (12.7) |  |
| Waist circumference, cm (mean (SD)) |  | 105.0 (12.9) | 109.8 (13.3) | <0.001 |
| At-risk AF, n (%) |  | 16 (6.6) | 40 (11.3) | 0.104 |
| T2DM, n (%) |  | 102 (42.1) | 191 (54.1) | 0.005 |
| Hypertension, n (%) |  | 161 (66.5) | 242 (68.6) | 0.667 |
| Dyslipidaemia, n (%) |  | 154 (63.6) | 271 (76.8) | 0.001 |
| (History of) CVD, n (%) |  | 50 (20.7) | 110 (31.2) | 0.006 |
| MetS, n (%) |  | 167 (77.3) | 265 (81.0) | 0.328 |
| Laboratory measurements | | | | |
| Platelets, *10^9^ (mean (SD)) |  | 262.1 (63.7) | 265.9 (74.3) | 0.516 |
| AST, U/L (median (IQR)) |  | 26 (21-31) | 25 (20-33) | 0.961 |
| ALT, U/L (median (IQR)) |  | 27 (22-37) | 28 (20-44) | 0.555 |
| yGT, U/L (median (IQR)) |  | 27 (19-43) | 31 (21-49) | 0.003 |
| ALP, U/L (median (IQR)) |  | 78 (66-93) | 79 (65-95) | 0.879 |
| Albumin, g/L (median (IQR)) |  | 40 (39-43) | 40 (37-42) | <0.001 |
| Fasting glucose, mmol/L (median (IQR)) |  | 5.8 (5.2-7.0) | 5.9 (5.3-7.9) | 0.189 |
| HbA1c, mmol/mol (median (IQR)) |  | 43 (38-53) | 46 (39-62) | 0.002 |
| Total cholesterol, mmol/L (mean (SD)) |  | 4.9 (1.2) | 4.4 (1.4) | <0.001 |
| LDL, mmol/L (mean (SD)) |  | 2.8 (1.0) | 2.4 (1.2) | <0.001 |
| HDL, mmol/L (mean (SD)) |  | 1.3 (1.1-1.6) | 1.2 (1.0-1.4) | <0.001 |
| Triglycerides, mmol/L (median (IQR)) |  | 1.3 (1.0-2.1) | 1.4 (0.9-2.2) | 0.528 |

BMI, body mass index; T2DM, type 2 diabetes mellitus; CVD, cardiovascular disease; MetS, metabolic syndrome; AST, aspartate transaminase; ALT, alanine transaminase; yGT, gamma-glutamyltransferase; ALP, alkaline phosphatase; LDL, low-density lipoprotein; HDL, high-density lipoprotein.

### **Supplemental table 2.** Participant characteristics stratified for the presence of T2DM.

|  |  | **T2DM present** | **T2DM absent** | **p-value** |
| --- | --- | --- | --- | --- |
| n |  | 293 | 302 |  |
| Age, years (median (IQR)) |  | 61 (54-68) | 59 (48-67) | 0.002 |
| Sex, n women (%) |  | 121 (41.3) | 153 (51.7) | 0.013 |
| Healthcare line, n (%) | Primary care | 102 (34.8) | 140 (46.4) | <0.001 |
|  | Secondary care | 69 (23.5) | 95 (31.5) |  |
|  | Tertiary care | 122 (41.6) | 67 (22.2) |  |
| Weight, kg (mean (SD)) |  | 92.3 (18.3) | 91.0 (18.6) | 0.379 |
| BMI, kg/m^2^ (median (IQR)) |  | 30.0 (27.1-33.3) | 30.1 (26.8-33.8) | 0.841 |
| BMI categories, n (%) | <25.0 kg/m^2^ | 27 (9.2) | 37 (12.3) | 0.234 |
|  | 25.0-30.0 kg/m^2^ | 117 (39.9) | 109 (36.1) |  |
|  | 30.0-35.0 kg/m^2^ | 99 (33.8) | 98 (32.5) |  |
|  | ≥35.0 kg/m^2^ | 50 (17.1) | 58 (19.2) |  |
| Waist circumference, cm (mean (SD)) |  | 109.1 (13.6) | 106.7 (13.1) | 0.030 |
| At-risk AF, n (%) |  | 37 (12.6) | 19 (6.3) | 0.012 |
| Hypertension, n (%) |  | 226 (77.1) | 117 (58.6) | <0.001 |
| Dyslipidaemia, n (%) |  | 235 (80.2) | 190 (62.9) | <0.001 |
| (History of) CVD, n (%) |  | 101 (34.5) | 59 (19.5) | <0.001 |
| MetS, n (%) |  | 258 (93.8) | 174 (64.9) | <0.001 |
| Laboratory measurements | | | | |
| Platelets, *10^9^ (mean (SD)) |  | 261.8 (73.2) | 266.8 (67.2) | 0.389 |
| AST, U/L (median (IQR)) |  | 24 (20-31) | 27 (22-33) | <0.001 |
| ALT, U/L (median (IQR)) |  | 27 (20-38) | 29 (22-46) | 0.013 |
| yGT, U/L (median (IQR)) |  | 31 (21-47) | 29 (20-45) | 0.208 |
| AF, U/L (median (IQR)) |  | 81 (66-99) | 77 (65-91) | 0.038 |
| Albumin, g/L (median (IQR)) |  | 40 (38-42) | 40 (38-42) | 0.155 |
| Fasting glucose, mmol/L (median (IQR)) |  | 7.6 (6.4-9.9) | 5.4 (5.1-5.9) | <0.001 |
| HbA1c, mmol/mol (median (IQR)) |  | 58 (50-67) | 39 (36-43) | <0.001 |
| Total cholesterol, mmol/L (mean (SD)) |  | 4.2 (1.1) | 5.1 (1.3) | <0.001 |
| LDL, mmol/L (mean (SD)) |  | 2.1 (1.0) | 3.0 (1.2) | <0.001 |
| HDL, mmol/L (mean (SD)) |  | 1.2 (1.0-1.4) | 1.3 (1.1-1.6) | <0.001 |
| Triglycerides, mmol/L (median (IQR)) |  | 1.5 (1.0-2.2) | 1.3 (0.9-2.1) | 0.311 |

BMI, body mass index; T2DM, type 2 diabetes mellitus; CVD, cardiovascular disease; MetS, metabolic syndrome; AST, aspartate transaminase; ALT, alanine transaminase; yGT, gamma-glutamyltransferase; ALP, alkaline phosphatase; LDL, low-density lipoprotein; HDL, high-density lipoprotein.

### **Supplemental table 3.** Descriptive statistics non-invasive tests, stratified for the lines of care.

|  |  | **Primary care** | **Hospital care** | **p-value** |
| --- | --- | --- | --- | --- |
| n |  | 242 | 353 |  |
| Steatosis NITs | | |  |  |
| CAP, dB/m (median (IQR)) |  | 277 (235-325) | 302 (260-342) | <0.001 |
| CAP categories, n (%) | <248 dB/m | 79 (32.9) | 76 (21.5) | <0.001 |
|  | 248-260 dB/m | 20 (8.3) | 11 (3.1) |  |
|  | 260-290 dB/m | 36 (15.0) | 56 (15.9) |  |
|  | ≥290 dB/m | 105 (43.8) | 210 (59.5) |  |
| Fibrosis NITs | | |  |  |
| FIB4 (median (IQR)) |  | 1.17 (0.89-1.51) | 1.08 (0.79-1.44) | 0.018 |
| FIB4 categories, n (%) | <1.30 | 142 (61.2) | 217 (66.4) | 0.373 |
|  | 1.30-2.67 | 85 (36.6) | 106 (32.4) |  |
|  | ≥2.67 | 5 (2.2) | 4 (1.2) |  |
| MAF5 (median (IQR)) |  | 1.0 (-0.4-2.5) | 1.9 (0.6-3.1) | <0.001 |
| MAF5 categories, n (%) | <0 | 71 (30.6) | 43 (13.2) | <0.001 |
|  | 0-1 | 44 (19.0) | 58 (17.8) |  |
|  | ≥1 | 117 (50.4) | 224 (68.9) |  |
| NFS (mean (SD)) |  | -1.32 (1.26) | -1.15 (1.40) | 0.151 |
| NFS categories, n (%) | <-1.455 | 104 (45.4) | 127 (39.0) | 0.291 |
|  | -1.455-0.67 | 110 (48.0) | 172 (52.8) |  |
|  | ≥0.67 | 15 (6.6) | 27 (8.3) |  |
| LSM, kPa (median (IQR)) |  | 4.7 (3.8-5.9) | 5.7 (4.6-7.3) | <0.001 |
| LSM categories, n (%) | <8.0 kPa | 222 (91.7) | 285 (80.7) | 0.002 |
|  | 8.0-15.0 kPa | 16 (6.6) | 55 (15.6) |  |
|  | ≥15.0 kPa | 4 (1.7) | 13 (3.7) |  |
| ELF (median (IQR)) |  | 9.3 (8.9-9.7) | 9.1 (8.5-9.6) | 0.010 |
| ELF categories, n (%) | <7.7 | 7 (2.9) | 14 (4.2) | 0.722 |
|  | 7.7-9.8 | 184 (77.0) | 253 (75.3) |  |
|  | ≥9.8 | 48 (20.1) | 69 (20.5) |  |
| PRO-C3 (median (IQR)) |  | 11.3 (9.2-13.6) | 12.4 (10.1-16.2) | <0.001 |
| PRO-C3 categories, n (%) | <15.6 | 208 (87.4) | 235 (70.1) | <0.001 |
|  | ≥15.6 | 30 (12.6) | 100 (29.9) |  |
| PRO-C6 (median (IQR)) |  | 10.1 (8.6-12.3) | 9.4 (8.0-11.6) | 0.003 |
| PRO-C6 categories, n (%) | <12.0 | 169 (71.0) | 257 (76.7) | 0.123 |
|  | ≥12.0 | 69 (29.0) | 78 (23.3) |  |

NITs, non-invasive tests; CAP, controlled attenuation parameter; FIB4, fibrosis-4 index; MAF5, metabolic dysfunction–associated fibrosis-5 score; NFS, NAFLD-fibrosis score; LSM, liver stiffness measurement; ELF, enhanced liver fibrosis-test; PRO-C3, procollagen type III N-terminal propeptide; PRO-C6, procollagen type VI N-terminal propeptide.

### **Supplemental table 4.** Descriptive statistics non-invasive tests, stratified for the presence of T2DM.

|  |  | **T2DM present** | **T2DM absent** | **p-value** |
| --- | --- | --- | --- | --- |
| n |  | 293 | 302 |  |
| Steatosis NITs | | |  |  |
| CAP, dB/m (median (IQR)) |  | 299 (251-341) | 289 (240-334) | 0.068 |
| CAP categories, n (%) | <248 dB/m | 70 (24.1) | 85 (28.1) | 0.300 |
|  | 248-260 dB/m | 13 (4.5) | 18 (6.0) |  |
|  | 260-290 dB/m | 42 (14.4) | 50 (16.6) |  |
|  | ≥290 dB/m | 166 (57.0) | 149 (49.3) |  |
| Fibrosis NITs | | |  |  |
| FIB4 (median (IQR)) |  | 1.12 (0.82-1.50) | 1.09 (0.83-1.44) | 0.489 |
| FIB4 categories, n (%) | <1.30 | 173 (64.1) | 186 (64.4) | 0.198 |
|  | 1.30-2.67 | 90 (33.3) | 101 (34.9) |  |
|  | ≥2.67 | 7 (2.6) | 2 (0.7) |  |
| MAF5 (median (IQR)) |  | 2.5 (1.6-3.7) | 0.5 (-0.5-1.7) | <0.001 |
| MAF5 categories, n (%) | <0 | 13 (4.9) | 101 (34.9) | <0.001 |
|  | 0-1 | 28 (10.4) | 74 (25.6) |  |
|  | ≥1 | 227 (84.7) | 114 (39.4) |  |
| NFS (mean (SD)) |  | -0.51 (1.17) | -1.88 (1.16) | <0.001 |
| NFS categories, n (%) | <-1.455 | 56 (20.8) | 175 (61.2) | <0.001 |
|  | -1.455-0.67 | 173 (64.3) | 109 (38.1) |  |
|  | ≥0.67 | 40 (14.9) | 2 (0.7) |  |
| LSM, kPa (median (IQR)) |  | 5.6 (4.2-7.5) | 5.1 (4.2-6.3) | 0.002 |
| LSM categories, n (%) | <8.0 kPa |  |  |  |
|  | 8.0-15.0 kPa | 45 (15.4) | 26 (8.6) |  |
|  | ≥15.0 kPa | 14 (4.8) | 3 (1.0) |  |
| ELF (median (IQR)) |  | 9.3 (8.8-9.8) | 9.0 (8.5-9.6) | <0.001 |
| ELF categories, n (%) | <7.7 | 7 (2.5) | 14 (4.8) | 0.092 |
|  | 7.7-9.8 | 210 (74.2) | 227 (77.7) |  |
|  | ≥9.8 | 66 (23.3) | 51 (17.5) |  |
| PRO-C3 (median (IQR)) |  | 12.0 (9.7-15.7) | 12.0 (9.8-14.6) | 0.702 |
| PRO-C3 categories, n (%) | <15.6 | 210 (74.2) | 233 (80.3) | 0.079 |
|  | ≥15.6 | 73 (25.8) | 57 (19.7) |  |
| PRO-C6 (median (IQR)) |  | 9.7 (8.1-12.7) | 9.6 (8.1-11.6) | 0.196 |
| PRO-C6 categories, n (%) | <12.0 | 195 (68.9) | 231 (79.7) | 0.061 |
|  | ≥12.0 | 88 (31.1) | 59 (20.3) |  |

NITs, non-invasive tests; CAP, controlled attenuation parameter; FIB4, fibrosis-4 index; MAF5, metabolic dysfunction–associated fibrosis-5 score; NFS, NAFLD-fibrosis score; LSM, liver stiffness measurement; ELF, enhanced liver fibrosis-test; PRO-C3, procollagen type III N-terminal propeptide; PRO-C6, procollagen type VI N-terminal propeptide; T2DM, type 2 diabetes mellitus.

**Supplemental table 5**. Distribution of criteria contributing to at-risk classification in the active study arm and the regular care comparator arm.

|  | According to flow chart, n | In total, n (%) |
| --- | --- | --- |
| Active study arm | | |
| Total correct referral to hepatology | 56 | 56 (100.0) |
| Second LSM ≥8.0 kPa | 18 | 38 (67.9) |
| First LSM ≥15.0 kPa | 11 | 17 (30.4) |
| Liver biopsy ≥F2 | 14 | 14 (25.0) |
| Regular care comparator arm | | |
| Total correct referral to hepatology | 65 | 65 (100.0) |
| Second LSM ≥8.0 kPa | 23 | 45 (69.2) |
| First LSM ≥15.0 kPa | 12 | 17 (26.2) |
| Liver biopsy ≥F2 | 23 | 23 (35.4) |

LSM, liver stiffness measurement.

### **Supplemental table 6.** Diagnostic accuracy results and performance of individual NITs for at-risk advanced fibrosis.

|  | Stratified as at low-risk, % | Stratified as at intermediate-risk, % | Stratified as at high-risk, % | AUC-ROC (95% CI) | Cut-off | PPV (95% CI) | NPV (95% CI) | Sensitivity (95% CI) | Specificity (95% CI) | NNS, n |
| --- | --- | --- | --- | --- | --- | --- | --- | --- | --- | --- |
| FIB4 | 64.2 | 34.2 | 1.6 | 0.58 (0.49-0.67) | 1.30 | 13.0 (8.3-17.7) | 92.2 (89.4-95.0) | 48.1 (34.8-61.5) | 65.5 (61.4-69.7) | 21.5 |
|  |  |  |  |  | 2.67 | 66.7 (35.9-97.5) | 91.3 (88.9-93.6) | 11.1 (2.7-19.5) | 99.4 (98.7-100.0) | 93.2 |
| MAF5 | 20.5 | 18.3 | 61.2 | 0.79 (0.73-0.86) | 0.0 | 11.7 (8.7-14.7) | 99.1 (97.4-100.0) | 98.1 (94.5-100.0) | 22.4 (18.8-26.1) | 10.7 |
|  |  |  |  |  | 1.0 | 13.8 (10.1-17.4) | 97.2 (95.0-99.4) | 88.7 (80.1-97.2) | 41.7 (37.4-46.0) | 11.9 |
| NFS | 41.6 | 50.8 | 7.6 | 0.70 (0.63-0.78) | -1.455 | 13.6 (9.8-17.3) | 96.1 (93.6-98.6) | 83.0 (72.9-93.1) | 44.2 (39.9-48.6) | 12.6 |
|  |  |  |  |  | 0.67 | 28.6 (14.9-42.2) | 92.0 (89.7-94.4) | 22.6 (11.4-33.9) | 94.0 (92.0-96.1) | 46.3 |
| VCTE | 85.2 | NA | 14.8 | 0.97 (0.95-0.99) | 8.0 | 61.4 (51.2-71.5) | 99.6 (99.1-100.0) | 96.4 (91.6-100.0) | 93.7 (91.6-95.7) | 11.0 |
| ELF | 3.7 | 76.0 | 20.3 | 0.60 (0.52-0.69) | 7.7 | 9.7 (7.3-12.2) | 95.2 (86.1-100.0) | 98.2 (94.7-100.0) | 3.8 (2.2-5.5) | 10.6 |
|  |  |  |  |  | 9.8 | 16.2 (9.6-22.9) | 92.1 (89.7-94.6) | 34.5 (22.0-47.1) | 81.2 (77.8-84.5) | 30.3 |
| PRO-C3 | 77.3 | NA | 22.7 | 0.69 (0.61-0.76 | 15.6 | 21.5 (14.5-28.6) | 93.7 (91.4-95.9) | 50.0 (36.9-63.1) | 80.3 (76.8-83.7) | 20.5 |
| PRO-C6 | 74.3 | NA | 25.7 | 0.57 (0.48-0.65) | 9.35 | 13.6 (0.81-19.1) | 91.5 (88.9-94.2) | 35.7 (23.2-48.3) | 75.4 (71.7-79.1) | 28.7 |
| FAST | 79.6 | NA | 20.4 | 0.84 (0.78-0.90) | 0.35 | 31.3 (22.8-39.8) | 95.8 (93.9-97.6) | 65.5 (52.9-78.0) | 84.5 (81.3-87.6) | 15.7 |
| ADAPT | 73.0 | NA | 27.0 | 0.69 (0.61-0.77) | 6.3 | 19.7 (13.4-26.1) | 93.9 (91.6-96.2) | 54.5 (41.4-67.7) | 76.0 (72.3-79.7) | 19.7 |

NITs, non-invasive tests; CAP, controlled attenuation parameter; FIB4, fibrosis-4 index; MAF5, metabolic dysfunction–associated fibrosis-5 score; NFS, NAFLD-fibrosis score; VCTE, vibration-controlled transient elastography; ELF, enhanced liver fibrosis-test; PRO-C3, procollagen type III N-terminal propeptide; PRO-C6, procollagen type VI N-terminal propeptide; FAST, FiboScan-AST; AUC-ROC, area under the receiver operating characteristic curve; PPV, positive predictive value; NPV, negative predictive value; NNS, number-needed-to-screen.

| **Supplemental table 7.** Diagnostic accuracy results and performance of individual NITs for at-risk advanced fibrosis, primary care. | | | | | | | | | | |
| --- | --- | --- | --- | --- | --- | --- | --- | --- | --- | --- |
|  | Stratified as at low-risk, % | Stratified as at intermediate-risk, % | Stratified as at high-risk, % | AUC-ROC (95% CI) | Cut-off | PPV (95% CI) | NPV (95% CI) | Sensitivity (95% CI) | Specificity (95% CI) | NNS, n |
| Primary care | | | | | | | | | | |
| FIB4 | 61.2 | 36.6 | 2.2 | 0.60 (0.41-0.78) | 1.30 | 8.9 (3.0-14.8) | 95.1 (91.5-98.6) | 53.3 (28.1-78.6) | 62.2 (55.8-68.7) | 29.0 |
|  |  |  |  |  | 2.67 | 40.0 (0.0-82.9) | 94.3 (91.3-97.3) | 13.3 (0.0-30.5) | 98.6 (97.1-100.0) | 116.0 |
| MAF5 | 30.6 | 19.0 | 50.4 | 0.86 (0.76-0.96) | 0.0 | 9.3 (4.8-13.8) | 100.0 (100.0-100.0) | 100.0 (100.0-100.0) | 32.7 (26.5-39.0) | 15.5 |
|  |  |  |  |  | 1.0 | 11.1 (5.4-16.8) | 98.3 (95.9-100.0) | 86.7 (69.5-100.0) | 52.1 (45.4-58.7) | 17.8 |
| NFS | 45.4 | 48.0 | 6.6 | 0.75 (0.62-0.89) | -1.455 | 9.6 (4.4-14.8) | 97.1 (93.9-100.0) | 80.0 (59.8-100.0) | 47.2 (40.5-53.9) | 19.1 |
|  |  |  |  |  | 0.67 | 33.3 (9.5-57.2) | 95.3 (92.5-98.2) | 33.3 (9.5-57.2) | 95.3 (92.5-98.2) | 45.8 |
| VCTE | 91.7 | NA | 8.3 | 0.99 (0.99-1.00) | 8.0 | 80.0 (62.5-97.5) | 100.0 (100.0-100.0) | 100.0 (100.0-100.0) | 98.2 (96.5-99.9) | 15.1 |
| ELF | 2.9 | 77.0 | 20.1 | 0.51 (0.35-0.68) | 7.7 | 6.5 (3.3-9.6) | 85.7 (59.8-100.0) | 93.8 (81.9-100.0) | 2.7 (0.6-4.8) | 15.9 |
|  |  |  |  |  | 9.8 | 8.3 (0.5-16.2) | 93.7 (90.3-97.2) | 25.0 (3.8-46.2) | 80.3 (75.0-85.5) | 59.8 |
| PRO-C3 | 87.4 | NA | 12.6 | 0.82 (0.74-0.91 | 15.6 | 23.3 (8.2-38.5) | 95.7 (92.9-98.4) | 43.8 (19.4-68.1) | 89.6 (85.6-93.6) | 34.0 |
| PRO-C6 | 71.0 | NA | 29.0 | 0.49 (0.36-0.62) | 12.0 | 5.8 (0.3-11.3) | 92.9 (89.0-96.8) | 25.0 (3.8-446.2) | 70.7 (64.7-76.7) | 59.9 |

NITs, non-invasive tests; CAP, controlled attenuation parameter; FIB4, fibrosis-4 index; MAF5, metabolic dysfunction–associated fibrosis-5 score; NFS, NAFLD-fibrosis score; VCTE, vibration-controlled transient elastography; ELF, enhanced liver fibrosis-test; PRO-C3, procollagen type III N-terminal propeptide; PRO-C6, procollagen type VI N-terminal propeptide; AUC-ROC, area under the receiver operating characteristic curve; PPV, positive predictive value; NPV, negative predictive value; NNS, number-needed-to-screen.

### **Supplemental table 8.** Diagnostic accuracy results and performance of individual NITs for at-risk advanced fibrosis, hospital outpatient clinic care.

|  | Stratified as at low-risk, % | Stratified as at intermediate-risk, % | Stratified as at high-risk, % | AUC-ROC (95% CI) | Cut-off | PPV (95% CI) | NPV (95% CI) | Sensitivity (95% CI) | Specificity (95% CI) | NNS, n |
| --- | --- | --- | --- | --- | --- | --- | --- | --- | --- | --- |
| Hospital care | | | | | | | | | | |
| FIB4 | 66.4 | 32.4 | 1.2 | 0.59 (0.49-0.69) | 1.30 | 16.4 (9.5-23.3) | 90.3 (86.4-94.3) | 46.2 (30.5-61.8) | 68.1 (62.7-73.4) | 18.2 |
|  |  |  |  |  | 2.67 | 100.0 (100.0-100.0) | 89.2 (85.8-92.6) | 10.3 (0.7-19.8) | 100.0 (100.0-100.0) | 81.8 |
| MAF5 | 13.2 | 17.8 | 68.9 | 0.70 (0.66-0.84) | 0.0 | 13.1 (9.2-17.1) | 97.7 (93.2-100.0) | 97.4 (92.3-100.0) | 14.6 (10.5-18.7) | 8.8 |
|  |  |  |  |  | 1.0 | 15.2 (10.5-19.9) | 96.0 (92.2-99.8) | 89.5 (79.7-99.2) | 33.8 (28.3-39.3) | 9.6 |
| NFS | 39.0 | 52.8 | 8.3 | 0.67 (0.59-0.76) | -1.455 | 16.1 (11.0-21.2) | 95.3 (91.6-99.0) | 84.2 (72.6-95.8) | 42.0 (36.3-47.7) | 10.2 |
|  |  |  |  |  | 0.67 | 25.9 (9.4-42.5) | 89.6 (86.2-93.1) | 18.4 (6.1-30.7) | 93.1 (90.1-96.0) | 12.1 |
| VCTE | 80.7 | NA | 28.1 | 0.95 (0.92-0.98) | 8.0 | 80.0 (62.5-97.5) | 100.0 (100.0-100.0) | 100.0 (100.0-100.0) | 98.2 (97.0-100) | 9.3 |
| ELF | 4.2 | 75.3 | 20.5 | 0.66 (0.57-0.75) | 7.7 | 12.1 (8.5-15.7) | 100.0 (100.0-100.0) | 100.0 (100.0-100.0) | 4.7 (2.3-7.1) | 8.6 |
|  |  |  |  |  | 9.8 | 21.7 (12.0-31.5) | 91.0 (87.6-94.4) | 38.5 (23.2-53.7) | 81.8 (77.4-86.2) | 22.4 |
| PRO-C3 | 70.1 | NA | 29.9 | 0.62 (0.51-0.72) | 15.6 | 21.0 (13.0-29.0) | 91.9 (8.8-95.4) | 52.5 (37.0-68.0) | 73.2 (68.2-78.3) | 16.0 |
| PRO-C6 | 76.7 | NA | 23.3 | 0.59 (0.49-0.7) | 12.0 | 20.5 (11.6-29.5) | 90.7 (87.1-94.2) | 40.0 (24.8-55.2) | 79.0 (74.3-83.6) | 20.9 |

NITs, non-invasive tests; CAP, controlled attenuation parameter; FIB4, fibrosis-4 index; MAF5, metabolic dysfunction–associated fibrosis-5 score; NFS, NAFLD-fibrosis score; VCTE, vibration-controlled transient elastography; ELF, enhanced liver fibrosis-test; PRO-C3, procollagen type III N-terminal propeptide; PRO-C6, procollagen type VI N-terminal propeptide; AUC-ROC, area under the receiver operating characteristic curve; PPV, positive predictive value; NPV, negative predictive value; NNS, number-needed-to-screen.

### **Supplemental table 9.** Diagnostic results and performance of individual NITs for at-risk advanced fibrosis, T2DM present.

|  | Stratified as at low-risk, % | Stratified as at intermediate-risk, % | Stratified as at high-risk, % | AUC-ROC (95% CI) | Cut-off | PPV (95% CI) | NPV (95% CI) | Sensitivity (95% CI) | Specificity (95% CI) | NNS, n |
| --- | --- | --- | --- | --- | --- | --- | --- | --- | --- | --- |
| T2DM present | | | | | | | | | | |
| FIB4 | 64.1 | 33.3 | 2.6 | 0.60 (0.48-0.71) | 1.30 | 18.6 (10.8-26.3) | 90.3 (86.0-94.7) | 51.4 (34.9-68.0) | 66.8 (60.8-72.8) | 15.0 |
|  |  |  |  |  | 2.67 | 71.4 (38.0-100.0) | 88.6 (84.8-92.4) | 14.3 (2.7-25.9) | 99.1 (98.0-100.0) | 54.0 |
| MAF5 | 4.9 | 10.4 | 84.7 | 0.75 (0.65-0.85) | 0.0 | 13.3 (9.2-17.5) | 100.0 (100.0-100.0) | 100.0 (100.0-100.0) | 5.6 (2.6-8.5) | 7.9 |
|  |  |  |  |  | 1.0 | 14.1 (9.6-18.6) | 95.1 (88.5-100.0) | 94.1 (86.2-100.0) | 16.7 (11.9-21.4) | 8.4 |
| NFS | 20.8 | 64.3 | 14.9 | 0.67 (0.58-0.76) | -1.455 | 16.0 (11.0-20.9) | 98.2 (94.7-100.0) | 97.1 (91.6-100.0) | 23.5 (18.1-28.9) | 7.9 |
|  |  |  |  |  | 0.67 | 27.5 (13.7-41.3) | 89.5 (85.6-93.5) | 31.4 (16.0-46.8) | 87.6 (83.4-91.8) | 24.5 |
| VCTE | 79.9 | NA | 20.1 | 0.97 (0.96-0.99) | 8.0 | 61.0 (48.6-73.5) | 99.6 (98.7-100.0) | 97.3 (92.1-100.0) | 91.0 (87.5-94.5) | 8.1 |
| ELF | 2.5 | 74.2 | 23.3 | 0.62 (0.53-0.72) | 7.7 | 13.4 (9.4-17.4) | 100.0 (100.0-100.0) | 100.0 (100.0-100.0) | 2.8 (0.8-4.9) | 7.6 |
|  |  |  |  |  | 9.8 | 21.2 (11.3-31.1) | 89.4 (85.3-93.5) | 37.8 (22.2-53.5) | 78.9 (73.8-84.0) | 20.0 |
| PRO-C3 | 74.2 | NA | 25.8 | 0.65 (0.55-0.76) | 15.6 | 23.3 (13.6-33.0) | 90.5 (86.5-94.4) | 45.9 (29.9-62.0) | 77.2 (72.0-82.5) | 16.6 |
| PRO-C6 | 68.9 | NA | 31.1 | 0.59 (0.48-0.69) | 12.0 | 18.2 (10.1-26.2) | 89.2 (84.9-93.6) | 43.2 (27.3-59.2) | 70.7 (65.0-76.4) | 17.7 |

NITs, non-invasive tests; CAP, controlled attenuation parameter; FIB4, fibrosis-4 index; MAF5, metabolic dysfunction–associated fibrosis-5 score; NFS, NAFLD-fibrosis score; VCTE, vibration-controlled transient elastography; ELF, enhanced liver fibrosis-test; PRO-C3, procollagen type III N-terminal propeptide; PRO-C6, procollagen type VI N-terminal propeptide; AUC-ROC, area under the receiver operating characteristic curve; PPV, positive predictive value; NPV, negative predictive value; NNS, number-needed-to-screen; T2DM, type 2 diabetes mellitus.

### **Supplemental table 10.** Diagnostic results and performance of individual NITs for at-risk advanced fibrosis, T2DM absent.

|  | Stratified as at low-risk, % | Stratified as at intermediate-risk, % | Stratified as at high-risk, % | AUC-ROC (95% CI) | Cut-off | PPV (95% CI) | NPV (95% CI) | Sensitivity (95% CI) | Specificity (95% CI) | NNS, n |
| --- | --- | --- | --- | --- | --- | --- | --- | --- | --- | --- |
| T2DM absent | | | | | | | | | | |
| FIB4 | 64.4 | 34.9 | 0.7 | 0.55 (0.40-0.70) | 1.30 | 7.8 (2.6-12.9) | 94.1 (90.7-97.5) | 42.1 (19.9-64.3) | 64.8 (59.1-70.5) | 36.1 |
|  |  |  |  |  | 2.67 | 50.0 (0.0-100.0) | 93.7 (90.9-96.5) | 5.3 (0.0-15.3) | 99.6 (98.9-100.0) | 289.0 |
| MAF5 | 34.9 | 25.6 | 39.4 | 0.81 (0.71-0.92) | 0.0 | 9.6 (5.4-13.8) | 99.0 (97.1-100.0) | 94.7 (84.7-100.0) | 37.0 (31.3-42.8) | 16.1 |
|  |  |  |  |  | 1.0 | 13.2 (7.0-19.4) | 97.7 (95.5-99.9) | 78.9 (60.6-97.3) | 63.3 (57.6-69.1) | 19.3 |
| NFS | 61.2 | 38.1 | 0.7 | 0.66 (0.52-0.80) | -1.455 | 9.0 (3.7-14.3) | 95.4 (92.3-98.5) | 55.6 (32.6-78.5) | 62.3 (56.5-68.1) | 28.6 |
|  |  |  |  |  | 0.67 | 50.0 (0.0-100.0) | 94.0 (91.3-96.8) | 5.6 (0.0-16.1) | 99.6 (98.9-100.0) | 286.0 |
| VCTE | 90.4 | NA | 9.6 | 0.96 (0.91, 100.0) | 8.0 | 62.1 (44.4-79.7) | 99.6 (98.9-100.0) | 94.7 (84.7-100.0) | 96.1 (93.9-98.4) | 16.8 |
| ELF | 4.8 | 77.7 | 17.5 | 0.52 (0.37-0.68) | 7.7 | 6.1 (3.3-8.9) | 92.9 (79.4-100.0) | 94.4 (83.9-100.0) | 4.7 (2.2-7.3) | 17.2 |
|  |  |  |  |  | 9.8 | 9.8 (1.6-18.0) | 94.6 (91.8-97.5) | 27.8 (7.1-48.5) | 83.2 (78.8-87.6) | 58.4 |
| PRO-C3 | 80.3 | NA | 19.7 | 0.75 (0.64-0.86) | 15.6 | 19.3 (9.1-29.5) | 96.6 (94.2-98.9) | 57.9 (35.7-80.1) | 83.0 (78.6-87.5) | 26.4 |
| PRO-C6 | 79.7 | NA | 20.3 | 0.51 (0.39-0.63) | 12.0 | 6.8 (0.4-13.2) | 93.5 (90.3-96.7) | 21.1 (2.3-9.4) | 79.7 (74.84.5) | 72.5 |

NITs, non-invasive tests; CAP, controlled attenuation parameter; FIB4, fibrosis-4 index; MAF5, metabolic dysfunction–associated fibrosis-5 score; NFS, NAFLD-fibrosis score; VCTE, vibration-controlled transient elastography; ELF, enhanced liver fibrosis-test; PRO-C3, procollagen type III N-terminal propeptide; PRO-C6, procollagen type VI N-terminal propeptide; AUC-ROC, area under the receiver operating characteristic curve; PPV, positive predictive value; NPV, negative predictive value; NNS, number-needed-to-screen; T2DM, type 2 diabetes mellitus.

### **Supplemental table 11.** Spearman’s correlation non-invasive tests.

|  | FIB4 | | MAF5 | | NFS | | LSM | | ELF | | PRO-C3 | | PRO-C6 | |
| --- | --- | --- | --- | --- | --- | --- | --- | --- | --- | --- | --- | --- | --- | --- |
|  | R | p-value | R | p-value | R | p-value | R | p-value | R | p-value | R | p-value | R | p-value |
| FIB4 |  |  |  |  |  |  |  |  |  |  |  |  |  |  |
| MAF5 | 0.192 | <0.001 |  |  |  |  |  |  |  |  |  |  |  |  |
| NFS | 0.667 | <0.001 | 0.521 | <0.001 |  |  |  |  |  |  |  |  |  |  |
| LSM | -0.011 | 0.792 | 0.412 | <0.001 | 0.154 | <0.001 |  |  |  |  |  |  |  |  |
| ELF | 0.452 | <0.001 | 0.142 | <0.001 | 0.425 | <0.001 | 0.050 | 0.228 |  |  |  |  |  |  |
| PRO-C3 | -0.028 | 0.520 | 0.221 | <0.001 | 0.031 | 0.479 | 0.242 | <0.001 | 0.169 | <0.001 |  |  |  |  |
| PRO-C6 | 0.064 | 0.131 | 0.069 | 0.110 | 0.077 | 0.074 | 0.033 | 0.429 | 0.259 | <0.001 | 0.262 | <0.001 |  |  |

NITs, non-invasive tests; FIB4, fibrosis-4 index; MAF5, metabolic dysfunction–associated fibrosis-5 score; NFS, NAFLD-fibrosis score; VCTE, vibration-controlled transient elastography; ELF, enhanced liver fibrosis-test; PRO-C3, procollagen type III N-terminal propeptide; PRO-C6, procollagen type VI N-terminal propeptide.

### **Supplemental table 12.** Agreement VCTE (LSM), ELF, PRO-C3 and PRO-C6, determined by Cohen’s kappa.

|  | LSM <8.0 kPa, n | LSM ≥8.0 kPa, n | ELF <9.8, n | ELF ≥9.8, n | PRO-C3 <15.6, n | PRO-C3 ≥15.6, n | PRO-C6 <12.0, n | PRO-C6 ≥12.0, n |
| --- | --- | --- | --- | --- | --- | --- | --- | --- |
| LSM <8.0 kPa |  |  | κ = 0.05 (95% CI: 0.00-0.09) | | κ = 0.13 (95% CI: 0.07-0.19) | | κ = 0.05 (95% CI: 0.00-0.11) | |
| LSM ≥8.0 kPa |  |  |  |  |  |  |  |  |
| ELF <9.8 | 405 | 59 |  |  | κ = 0.12 (95% CI: 0.06-0.17) | | κ = 0.08 (95% CI: 0.03-0.14) | |
| ELF ≥9.8 | 89 | 24 |  |  |  |  |  |  |
| PRO-C3 <15.6 | 396 | 94 | 374 | 85 |  |  | κ = 0.13 (95% CI: 0.07-0.18) | |
| PRO-C3 ≥15.6 | 47 | 36 | 68 | 44 |  |  |  |  |
| PRO-C6 <12.0 | 373 | 117 | 356 | 103 | 352 | 91 |  |  |
| PRO-C6 ≥12.0 | 55 | 28 | 41 | 41 | 76 | 54 |  |  |

VCTE, vibration-controlled transient elastography; LSM, liver stiffness measurement; ELF, enhanced liver fibrosis-test; PRO-C3, procollagen type III N-terminal propeptide; PRO-C6, procollagen type VI N-terminal propeptide.

### **Supplemental table 13.** Participant characteristics comparative arm.

|  |  | Entire comparative arm population |
| --- | --- | --- |
| n |  | 465 |
| Age, years (median (IQR)) |  | 50 (38-58) |
| Sex, n women (%) |  | 194 (41.8) |
| Referring healthcare line, n (%) | Primary care | 199 (42.8) |
|  | Secondary care | 54 (11.6) |
|  | Tertiary care | 212 (45.6) |
| BMI, kg/m^2^ (median (IQR)) |  | 29.6 (26.5-33.5) |
| T2DM, n (%) |  | 112 (24.0) |
| Hypertension, n (%) |  | 179 (38.5) |
| Dyslipidaemia, n (%) |  | 271 (58.2) |
| (History of) CVD, n (%) |  | 89 (19.1) |
| VCTE data | | |
| CAP, dB/m (median (IQR)) |  | 310 (268-352) |
| CAP categories, n (%) | <248 dB/m | 71 (16.9) |
|  | 248-260 dB/m | 22 (5.2) |
|  | 260-290 dB/m | 55 (13.1) |
|  | ≥290 dB/m | 272 (64.8) |
| LSM, kPa (median (IQR)) |  | 5.7 (4.7-7.6) |
| LSM categories, n (%) | <8.0 kPa | 37 (79.9) |
|  | 8.0-15.0 kPa | 76 (16.3) |
|  | ≥15.0 kPa | 18 (3.8) |
| Clinical reference standard | | |
| At-risk of advanced fibrosis (%) | Entire study population | 65 (14.0) |
|  | Primary care | 25 (12.8) |
|  | Secondary care | 16 (10.7) |
|  | Tertiary care | 34 (16.3) |

BMI, body mass index; T2DM, type 2 diabetes mellitus; CVD, cardiovascular disease; CAP, controlled attenuation parameter; VCTE, vibration-controlled transient elastography; LSM, liver stiffness measurement.

**Supplementary table 14.** Predictors of at-risk advanced MASLD fibrosis stratified for lines of care.

|  |  | Univariate regression | | Multivariate regression | |
| --- | --- | --- | --- | --- | --- |
|  | n (%) | OR | p-value | OR | p-value |
| Primary care | | | | | |
| T2DM | 102 (42.1) | 4.2 (1.5-14.3) | 0.007 | 3.9 (1.2-15.0) | 0.029 |
| Hypertension | 161 (66.5) | 2.5 (0.3-327.0) | 0.465 | NE | 0.992 |
| Dyslipidaemia | 154 (63.6) | 0.5 (0.2-1.5) | 0.238 | 0.5 (0.1-1.4) | 0.168 |
| Obesity | 97 (40.0) | 18.1 (4.4-166.6) | <0.001 | 21.4 (4.1-392.3) | 0.004 |
| Metabolic syndrome | 167 (77.3) | 10.1 (1.3-1293.0) | 0.020 | - | - |
| Hospital outpatient care | | | | | |
| T2DM | 191 (54.1) | 1.5 (0.8-2.9) | 0.265 | 1.7 (0.8-3.6) | 0.152 |
| Hypertension | 242 (68.6) | 1.2 (0.4-4.6) | 0.752 | 1.3 (0.4-5.7) | 0.722 |
| Dyslipidaemia | 271 (76.8) | 0.8 (0.4-1.6) | 0.461 | 0.8 (0.4-1.9) | 0.590 |
| Obesity | 208 (58.9) | 4.2 (1.9-11.0) | <0.001 | 4.8 (2.1-13.1) | 0.001 |
| Metabolic syndrome | 265 (81.0) | 0.9 (0.4-2.1) | 0.722 | - | - |

OR, odds ratio; T2DM, type 2 diabetes mellitus; NE, not estimable.

### **Supplemental table 15.** Diagnostic results and performance two-tiered care pathways using FAST and ADAPT.

|  | 2^nd^ test needed, % | Referral to hepatology, % | PPV, % (95% CI) | NPV, % (95% CI) | Sensitivity, % (95% CI) | Specificity, % (95% CI) | Correct referral rate, % | Improved correct referral rate, RR (95% CI) | NNS for correct referral, whole care path, n | NNS for correct referral, after 2^nd^ test, n |
| --- | --- | --- | --- | --- | --- | --- | --- | --- | --- | --- |
| Two-tiered care paths FAST, cutoff 0.35 | | | | | | | | | | |
| FIB4/FAST if FIB4 ≥1.30 | 35.8 | 9.3 | 42.3 (28.9-55.7) | 93.7 (91.6-95.8) | 40.7 (27.6-53.8) | 94.1 (92.0-96.1) | 42.3 | 3.02 (1.5-6.0) | 25.4 | 9.1 |
| MAF5/FAST if MAF5 ≥1.0 | 61.2 | 18.7 | 30.8 (21.9-39.6) | 95.4 (93.4-97.3) | 60.4 (47.2-73.5) | 85.7 (82.7-88.8) | 30.8 | 2.20 (1.1-4.5) | 17.4 | 10.7 |
| NFS/FAST if NFS ≥-1.455 | 58.4 | 12.3 | 44.1 (32.3-55.9) | 95.3 (93.4-97.2) | 56.6 (43.3-69.9) | 92.4 (90.1-94.7) | 44.1 | 3.15 (1.6-6.3) | 18.5 | 10.8 |
| Two-tiered care paths ADAPT, cutoff 6.3 | | | | | | | | | | |
| FIB4/ADAPT if FIB4 ≥1.30 | 36.0 | 15.3 | 24.7 (15.5-33.9) | 93.0 (90.6-95.3) | 38.9 (25.9-51.9) | 87.2 (84.3-90.1) | 24.7 | 1.76 (0.9-3.6) | 26.4 | 9.5 |
| MAF5/ADAPT if MAF5 ≥1.0 | 62.2 | 22.8 | 21.6 (14.4-28.8) | 93.9 (91.6-96.1) | 50.9 (37.5-64.4) | 80.2 (76.7-83.7) | 21.6 | 1.54 (0.7-3.2) | 20.3 | 12.6 |
| NFS/ADAPT if NFS ≥-1.455 | 59.2 | 24.3 | 21.8 (14.8-28.8) | 94.2 (92.0-96.5) | 54.7 (41.3-68.1) | 78.9 (75.4-82.5) | 21.8 | 1.56 (0.7-3.3) | 18.9 | 11.2 |

FIB4, fibrosis-4 index; MAF5, metabolic dysfunction–associated fibrosis-5 score; NFS, NAFLD-fibrosis score; FAST, FibroScan-AST; PPV, positive predictive value; NPV, negative predictive value; NNS, number-needed-to-screen; T2DM, type 2 diabetes mellitus.

### **Supplemental table 16.** Diagnostic results and performance two-tiered care pathways using VCTE, stratified for the lines of care.

|  | VCTE needed, % | Referral to hepatology, % | PPV, % (95% CI) | NPV, % (95% CI) | Sensitivity, % (95% CI) | Specificity, % (95% CI) | Correct referral rate, % | Improved correct referral rate, RR (95% CI) | NNS for correct referral, whole care path, n | NNS for correct referral, after VCTE, n |
| --- | --- | --- | --- | --- | --- | --- | --- | --- | --- | --- |
| Primary care | | | | | | | | | | |
| FIB4/VCTE if FIB4 ≥1.30 | 38.8 | 4.7 | 72.7 (46.4-99.0) | 96.8 (94.5-99.1) | 53.3 (28.1-78.6) | 98.6 (97.1-100.0) | 72.7 | 5.7 (2.7-11.8) | 29.0 | 11.3 |
| MAF5/VCTE if MAF5 ≥1.0 | 50.4 | 7.3 | 76.5 (56.3-96.6) | 99.1 (97.8-100.0) | 86.7 (69.5-100.0) | 98.2 (96.4-99.9) | 76.5 | 6.0 (2.8-12.6) | 17.8 | 9.0 |
| NFS/VCTE if NFS  ≥-1.455 | 54.6 | 7.0 | 75.0 (53.8-96.2) | 98.6 (97.0-100.0) | 80.0 (59.8-100.0) | 98.1 (96.3-99.9) | 75.0 | 5.9 (2.8-12.3) | 19.1 | 10.4 |
| Hospital care | | | | | | | | | | |
| FIB4/VCTE if FIB4 ≥1.30 | 33.6 | 7.6 | 68.0 (49.7-86.3) | 92.7 (89.8-95.6) | 43.6 (28.0-59.2) | 97.2 (95.3-99.1) | 68.0 | 4.5 (2.3-9.0) | 19.2 | 6.5 |
| MAF5/VCTE if MAF5 ≥1.0 | 68.9 | 17.2 | 58.9 (46.0-71.8) | 98.1 (96.5-99.8) | 86.8 (76.1-97.6) | 92.0 (88.8-95.1) | 58.9 | 3.9 (2.0-7.7) | 9.8 | 6.8 |
| NFS/VCTE if NFS  ≥-1.455 | 61.0 | 14.4 | 66.0 (52.4-79.5) | 97.5 (95.7-99.3) | 81.6 (69.3-93.9) | 94.4 (91.8-97.1) | 66.0 | 4.4 (2.2-8.7) | 10.5 | 6.4 |

FIB4, fibrosis-4 index; MAF5, metabolic dysfunction–associated fibrosis-5 score; NFS, NAFLD-fibrosis score; VCTE, vibration-controlled transient elastography; PPV, positive predictive value; NPV, negative predictive value; NNS, number-needed-to-screen.

### **Supplemental table 17.** Diagnostic results and performance two-tiered care pathways using VCTE, stratified for the presence of T2DM.

|  | VCTE needed, % | Referral to hepatology, % | PPV, % (95% CI) | NPV, % (95% CI) | Sensitivity, % (95% CI) | Specificity, % (95% CI) | Correct referral rate, % | Improved correct referral rate, RR (95% CI) | NNS for correct referral, whole care path, n | NNS for correct referral, after VCTE, n |
| --- | --- | --- | --- | --- | --- | --- | --- | --- | --- | --- |
| T2DM present | | | | | | | | | | |
| FIB4/VCTE if FIB4 ≥1.30 | 35.9 | 9.6 | 65.4 (47.1-83.7) | 92.6 (89.3-95.9) | 48.6 (32.0-65.1) | 96.2 (93.7-98.6) | 65.4 | 2.1 (1.2-3.8) | 15.9 | 5.7 |
| MAF5/VCTE if MAF5 ≥1.0 | 84.7 | 18.3 | 63.3 (49.8-76.8) | 98.6 (97.1-100.0) | 91.2 (81.6-100.0) | 92.3 (88.9-95.7) | 63.3 | 2.0 (1.1-3.6) | 8.6 | 7.3 |
| NFS/VCTE if NFS  ≥-1.455 | 79.2 | 19.0 | 64.7 (51.6-77.8) | 99.1 (97.8-100.0) | 94.3 (86.6-100.0) | 92.3 (88.9-95.7) | 64.7 | 2.1 (1.1-3.7) | 8.2 | 6.5 |
| T2DM absent | | | | | | | | | | |
| FIB4/VCTE if FIB4 ≥1.30 | 35.6 | 3.5 | 80.0 (55.2-100.0) | 96.1 (93.8-98.3) | 42.1 (19.9-64.3) | 99.3 (98.2-100.0) | 80.0 | 9.2 (3.9-21.5) | 36.1 | 12.9 |
| MAF5/VCTE if MAF5 ≥1.0 | 39.4 | 8.3 | 62.5 (43.1-81.9) | 98.5 (97.0-100.0) | 78.9 (60.6-97.3) | 96.7 (94.5-98.8) | 62.5 | 7.2 (3.2-16.1) | 19.3 | 7.6 |
| NFS/VCTE if NFS  ≥-1.455 | 38.8 | 4.2 | 83.3 (62.2-100.0) | 97.1 (95.1-99.1) | 55.6 (32.6-78.5) | 99.3 (98.2-100.0) | 83.3 | 9.6 (4.0-22.9) | 28.6 | 11.1 |

FIB4, fibrosis-4 index; MAF5, metabolic dysfunction–associated fibrosis-5 score; NFS, NAFLD-fibrosis score; VCTE, vibration-controlled transient elastography; PPV, positive predictive value; NPV, negative predictive value; NNS, number-needed-to-screen; T2DM, type 2 diabetes mellitus.

### **Supplemental table 18.** Diagnostic results and performance two-tiered care pathways using PRO-C3.

|  | PRO-C3 needed, % | Referral to hepatology, % | PPV, % (95% CI) | NPV, % (95% CI) | Sensitivity, % (95% CI) | Specificity, % (95% CI) | Correct referral rate, % | Improved correct referral rate, RR (95% CI) | NNS for correct referral, whole care path, n | NNS for correct referral, after PRO-C3, n |
| --- | --- | --- | --- | --- | --- | --- | --- | --- | --- | --- |
| FIB4/PRO-C3 if FIB4 ≥1.30 | 35.1 | 8.3 | 30.4 (7.1-43.7) | 92.0 (89.6-94.3) | 25.9 (14.2-37.6) | 93.5 (91.3-95.6) | 30.4 | 2.17 (1.1-4.4) | 38.8 | 13.9 |
| MAF5/PRO-C3 if MAF5 ≥1.0 | 61.4 | 17.0 | 27.2 (18.1-36.3) | 93.8 (91.5-96.0) | 47.2 (33.7-60.6) | 86.3 (83.2-89.3) | 27.2 | 1.94 (0.9-4.0) | 21.6 | 13.3 |
| NFS/PRO-C3 if NFS  ≥-1.455 | 58.6 | 14.5 | 26.9 (17.1-36.8) | 93.1 (90.7-95.4) | 39.6 (26.5-52.8) | 88.3 (85.4-91.1) | 26.9 | 1.92 (0.9-3.9) | 25.7 | 15.0 |

FIB4, fibrosis-4 index; MAF5, metabolic dysfunction–associated fibrosis-5 score; NFS, NAFLD-fibrosis score; PRO-C3, procollagen type III N-terminal propeptide; PPV, positive predictive value; NPV, negative predictive value; NNS, number-needed-to-screen.

### **Supplemental table 19.** Diagnostic results and performance two-tiered care pathways using PRO-C3, stratified for the lines of care.

|  | PRO-C3 needed, % | Referral to hepatology, % | PPV, % (95% CI) | NPV, % (95% CI) | Sensitivity, % (95% CI) | Specificity, % (95% CI) | Correct referral rate, % | Improved correct referral rate, RR (95% CI) | NNS for correct referral, whole care path, n | NNS for correct referral, after PRO-C3, n |
| --- | --- | --- | --- | --- | --- | --- | --- | --- | --- | --- |
| Primary care | | | | | | | | | | |
| FIB4/PRO-C3 if FIB4 ≥1.30 | 39.1 | 4.3 | 40.0 (9.6-70.4) | 95.0 (92.1-97.9) | 26.7 (4.3-49.0) | 97.2 (95.0-99.4) | 40.0 | 3.13 (1.5-6.4) | 57.5 | 22.5 |
| MAF5/PRO-C3 if MAF5 ≥1.0 | 50.9 | 8.7 | 30.0 (9.9-50.1) | 95.7 (93.0-98.5) | 40.0 (15.2-64.8) | 93.5 (90.2-96.8) | 30.0 | 2.34 (1.1-4.8) | 38.3 | 19.5 |
| NFS/PRO-C3 if NFS  ≥-1.455 | 55.3 | 8.8 | 25.0 (6.0-44.0) | 95.1 (93.0-98.5) | 33.3 (15.2-64.8) | 92.9 (90.2-96.8) | 25.0 | 1.95 (0.9-4.1) | 45.2 | 25.0 |
| Hospital care | | | | | | | | | | |
| FIB4/PRO-C3 if FIB4 ≥1.30 | 33.0 | 11.1 | 27.8 (13.1-42.4) | 89.9 (86.5-93.4) | 25.6 (11.9-39.3) | 90.9 (87.5-94.2) | 27.8 | 2.17 (1.0-4.5) | 32.4 | 10.7 |
| MAF5/PRO-C3 if MAF5 ≥1.0 | 70.4 | 22.6 | 26.4 (16.2-36.6) | 92.3 (88.9-95.6) | 50.0 (34.1-65.9) | 81.1 (76.5-85.7) | 26.4 | 2.06 (1.0-4.3) | 16.7 | 11.8 |
| NFS/PRO-C3 if NFS  ≥-1.455 | 62.0 | 18.1 | 27.6 (16.1-39.1) | 91.6 (88.3-95.0) | 42.1 (26.4-57.8) | 85.2 (81.0-89.3) | 27.6 | 2.16 (1.0-4.5) | 20.1 | 12.4 |

FIB4, fibrosis-4 index; MAF5, metabolic dysfunction–associated fibrosis-5 score; NFS, NAFLD-fibrosis score; PRO-C3, procollagen type III N-terminal propeptide; PPV, positive predictive value; NPV, negative predictive value; NNS, number-needed-to-screen.

### **Supplemental table 20.** Diagnostic results and performance two-tiered care pathways using PRO-C3, stratified for the presence of T2DM.

|  | PRO-C3 needed, % | Referral to hepatology, % | PPV, % (95% CI) | NPV, % (95% CI) | Sensitivity, % (95% CI) | Specificity, % (95% CI) | Correct referral rate, % | Improved correct referral rate, RR (95% CI) | NNS for correct referral, whole care path, n | NNS for correct referral, after PRO-C3, n |
| --- | --- | --- | --- | --- | --- | --- | --- | --- | --- | --- |
| T2DM present | | | | | | | | | | |
| FIB4/PRO-C3 if FIB4 ≥1.30 | 36.2 | 10.4 | 32.1 (14.8-49.4) | 89.2 (85.2-93.1) | 25.7 (11.2-40.2) | 91.8 (88.3-95.4) | 32.1 | 1.02 (0.6-1.9) | 29.8 | 10.8 |
| MAF5/PRO-C3 if MAF5 ≥1.0 | 86.0 | 22.0 | 25.9 (14.6-37.1) | 90.8 (86.8-94.7) | 44.1 (27.4-60.8) | 81.3 (76.3-86.3) | 25.9 | 0.82 (0.4-1.5) | 17.6 | 15.1 |
| NFS/PRO-C3 if NFS  ≥-1.455 | 80.1 | 21.8 | 25.9 (14.6-37.1) | 90.4 (86.8-94.7) | 42.9 (27.4-60.8) | 81.4 (76.4-86.4) | 25.9 | 0.82 (0.4-1.5) | 17.7 | 14.2 |
| T2DM absent | | | | | | | | | | |
| FIB4/PRO-C3 if FIB4 ≥1.30 | 36.0 | 6.3 | 27.8 (7.1-48.5) | 94.8 (92.1-97.4) | 26.3 (6.5-46.1) | 95.1 (92.5-97.7) | 27.8 | 0.88 (0.5-1.6) | 57.2 | 20.6 |
| MAF5/PRO-C3 if MAF5 ≥1.0 | 40.1 | 12.0 | 29.4 (14.1-44.7) | 96.4 (94.1-98.7) | 52.6 (30.2-75.1) | 87.5-(94.4) | 29.4 | 0.94 (0.5-1.7) | 28.4 | 11.4 |
| NFS/PRO-C3 if NFS  ≥-1.455 | 39.5 | 7.1 | 30.0 (9.9-50.1) | 95.4 (92.9-97.9) | 33.3 (11.6-55.1) | 94.7 (92.0-97.4) | 30.0 | 0.96 (0.5-1.7) | 46.8 | 18.5 |

FIB4, fibrosis-4 index; MAF5, metabolic dysfunction–associated fibrosis-5 score; NFS, NAFLD-fibrosis score; PRO-C3, procollagen type III N-terminal propeptide; PPV, positive predictive value; NPV, negative predictive value; NNS, number-needed-to-screen; T2DM, type 2 diabetes mellitus.

### **Supplemental table 21.** Diagnostic results and performance two-tiered care pathways using ELF.

|  | ELF needed, % | Referral to hepatology, % | PPV, % (95% CI) | NPV, % (95% CI) | Sensitivity, % (95% CI) | Specificity, % (95% CI) | Correct referral rate, % | Improved correct referral rate, RR (95% CI) | NNS for correct referral, whole care path, n | NNS for correct referral, after ELF, n |
| --- | --- | --- | --- | --- | --- | --- | --- | --- | --- | --- |
| FIB4/ELF if FIB4 ≥1.30 | 35.9 | 11.9 | 23.1 (13.9-32.8) | 91.9 (90.1-94.9) | 15.8 (21.2-46.7) | 89.8 (85.1-90.9) | 23.1 | 1.65 (0.8-3.4) | 36.4 | 13.1 |
| MAF5/ELF if MAF5 ≥1.0 | 60.8 | 14.2 | 23.4 (13.9-32.8) | 92.5 (90.1-94.9) | 34.0 (21.2-46.7) | 88.0 (85.1-90.9) | 23.4 | 1.67 (0.8-3.5) | 30.2 | 18.4 |
| NFS/ELF if NFS  ≥-1.455 | 58.3 | 16.4 | 20.2 (11.9-28.6) | 92.3 (89.8-94.7) | 34.0 (21.2-46.7) | 85.5 (82.4-88.6) | 20.2 | 1.44 (0.7-3.0) | 30.1 | 17.6 |

FIB4, fibrosis-4 index; MAF5, metabolic dysfunction–associated fibrosis-5 score; NFS, NAFLD-fibrosis score; ELF, enhanced liver fibrosis-test; PPV, positive predictive value; NPV, negative predictive value; NNS, number-needed-to-screen.

### **Supplemental table 22.** Diagnostic results and performance two-tiered care pathways using ELF, stratified for the lines of care.

|  | ELF needed, % | Referral to hepatology, % | PPV, % (95% CI) | NPV, % (95% CI) | Sensitivity, % (95% CI) | Specificity, % (95% CI) | Correct referral rate, % | Improved correct referral rate, RR (95% CI) | NNS for correct referral, whole care path, n | NNS for correct referral, after ELF, n |
| --- | --- | --- | --- | --- | --- | --- | --- | --- | --- | --- |
| Primary care | | | | | | | | | | |
| FIB4/ELF if FIB4 ≥1.30 | 39.1 | 11.3 | 11.5 (0.0-23.8) | 94.1 (90.9-97.3) | 20.0 (0.0-40.2) | 89.3 (85.2-93.4) | 11.5 | 0.9 (0.4-2.1) | 76.7 | 30.0 |
| MAF5/ELF if MAF5 ≥1.0 | 50.9 | 10.0 | 13.0 (0.0-26.8) | 94.2 (91.0-97.4) | 20.0 (0.0-40.2) | 90.7 (86.8-94.6) | 13.0 | 1.0 (0.4-2.3) | 76.7 | 39.0 |
| NFS/ELF if NFS  ≥-1.455 | 55.3 | 15.0 | 8.8 (0.0-18.4) | 93.8 (90.3-97.2) | 20.0 (0.0-40.2) | 85.3 (80.5-90.1) | 8.8 | 0.7 (0.3-1.7) | 75.3 | 41.7 |
| Hospital care | | | | | | | | | | |
| FIB4/ELF if FIB4 ≥1.30 | 33.8 | 12.0 | 28.2 (14.1-42.3) | 90.2 (86.8-93.7) | 28.2 (14.1-42.3) | 90.2 (86.8-93.7) | 28.2 | 1.9 (0.9-3.8) | 29.5 | 10.0 |
| MAF5/ELF if MAF5 ≥1.0 | 70.9 | 16.5 | 26.9 (14.9-39.0) | 91.3 (87.9-94.7) | 37.8 (22.2-53.5) | 86.4 (82.4-90.4) | 26.9 | 1.8 (0.9-3.6) | 22.6 | 16.0 |
| NFS/ELF if NFS  ≥-1.455 | 62.0 | 17.8 | 24.6 (13.4-35.7) | 90.9 (87.4-94.4) | 36.8 (21.5-52.2) | 84.8 (80.6-89.0) | 24.6 | 1.6 (0.8-3.3) | 22.9 | 14.2 |

FIB4, fibrosis-4 index; MAF5, metabolic dysfunction–associated fibrosis-5 score; NFS, NAFLD-fibrosis score; ELF, enhanced liver fibrosis-test; PPV, positive predictive value; NPV, negative predictive value; NNS, number-needed-to-screen.

### **Supplemental table 23.** Diagnostic results and performance two-tiered care pathways using ELF, stratified for the presence of T2DM.

|  | ELF needed, % | Referral to hepatology, % | PPV, % (95% CI) | NPV, % (95% CI) | Sensitivity, % (95% CI) | Specificity, % (95% CI) | Correct referral rate, % | Improved correct referral rate, RR (95% CI) | NNS for correct referral, whole care path, n | NNS for correct referral, after ELF, n |
| --- | --- | --- | --- | --- | --- | --- | --- | --- | --- | --- |
| T2DM present | | | | | | | | | | |
| FIB4/ELF if FIB4 ≥1.30 | 36.2 | 11.9 | 31.3 (15.2-47.3) | 89.4 (85.5-93.3) | 28.6 (13.6-43.5) | 90.6 (86.8-94.3) | 31.3 | 1.0 (0.5-1.8) | 26.8 | 9.7 |
| MAF5/ELF if MAF5 ≥1.0 | 86.3 | 19.8 | 23.1 (11.6-34.5) | 89.6 (85.4-93.7) | 35.3 (19.2-51.4) | 82.5 (77.6-87.5) | 23.1 | 0.7 (0.4-1.4) | 21.9 | 18.9 |
| NFS/ELF if NFS  ≥-1.455 | 80.4 | 21.1 | 23.2 (12.2-34.3) | 89.5 (85.3-93.6) | 37.1 (21.1-53.2) | 81.3 (76.3-86.3) | 23.2 | 0.7 (0.4-1.4) | 20.4 | 16.4 |
| T2DM absent | | | | | | | | | | |
| FIB4/ELF if FIB4 ≥1.30 | 35.9 | 11.5 | 14.7 (2.8-26.6) | 94.1 (91.2-97.0) | 25.0 (6.0-44.0) | 89.2 (85.5-92.9) | 14.7 | 1.7 (0.7-4.1) | 57.4 | 20.6 |
| MAF5/ELF if MAF5 ≥1.0 | 40.3 | 8.1 | 21.7 (4.9-38.6) | 95.0 (92.4-97.6) | 27.8 (7.1-48.5) | 93.2 (90.2-96.2) | 21.7 | 2.5 (1.1-5.8) | 56.6 | 22.8 |
| NFS/ELF if NFS  ≥-1.455 | 39.4 | 12.4 | 11.4 (0.9-22.0) | 94.3 (91.4-97.2) | 22.2 (3.0-41.4) | 88.3 (84.4-92.1) | 11.4 | 1.3 (0.5-3.3) | 70.5 | 27.8 |

FIB4, fibrosis-4 index; MAF5, metabolic dysfunction–associated fibrosis-5 score; NFS, NAFLD-fibrosis score; ELF, enhanced liver fibrosis-test; PPV, positive predictive value; NPV, negative predictive value; NNS, number-needed-to-screen; T2DM, type 2 diabetes mellitus.

### **Supplemental table 24.** Diagnostic results and performance two-tiered care pathways using PRO-C6.

|  | PRO-C6 needed, % | Referral to hepatology, % | PPV, % (95% CI) | NPV, % (95% CI) | Sensitivity, % (95% CI) | Specificity, % (95% CI) | Correct referral rate, % | Improved correct referral rate, RR (95% CI) | NNS for correct referral, whole care path, n | NNS for correct referral, after PRO-C6, n |
| --- | --- | --- | --- | --- | --- | --- | --- | --- | --- | --- |
| FIB4/PRO-C6 if FIB4 ≥1.30 | 35.1 | 9.5 | 20.8 (9.8-31.7) | 91.2 (88.7-93.7) | 20.4 (9.6-31.1) | 91.4 (88.9-93.9) | 20.8 | 1.48 (0.7-3.1) | 49.4 | 17.7 |
| MAF5/PRO-C6 if MAF5 ≥1.0 | 58.4 | 17.6 | 20.0 (12.0-28.0) | 92.4 (89.9-94.8) | 35.8 (22.9-48.8) | 84.4 (81.2-87.6) | 20.0 | 1.43 (0.7-3.0) | 28.5 | 16.6 |
| NFS/PRO-C6 if NFS  ≥-1.455 | 58.6 | 17.1 | 18.5 (10.5-26.4) | 91.9 (89.4-94.5) | 32.1 (19.5-44.6) | 84.6 (81.4-87.8) | 18.5 | 1.32 (0.6-2.8) | 31.7 | 18.6 |

FIB4, fibrosis-4 index; MAF5, metabolic dysfunction–associated fibrosis-5 score; NFS, NAFLD-fibrosis score; PRO-C6, procollagen type VI N-terminal propeptide; PPV, positive predictive value; NPV, negative predictive value; NNS, number-needed-to-screen.

### **Supplemental table 25.** Diagnostic results and performance two-tiered care pathways using PRO-C6, stratified for the lines of care.

|  | PRO-C6 needed, % | Referral to hepatology, % | PPV, % (95% CI) | NPV, % (95% CI) | Sensitivity, % (95% CI) | Specificity, % (95% CI) | Correct referral rate, % | Improved correct referral rate, RR (95% CI) | NNS for correct referral, whole care path, n | NNS for correct referral, after PRO-C6, n |
| --- | --- | --- | --- | --- | --- | --- | --- | --- | --- | --- |
| Primary care | | | | | | | | | | |
| FIB4/PRO-C6 if FIB4 ≥1.30 | 33.0 | 11.1 | 14.8 (1.4-28.2) | 94.6 (91.5-97.7) | 26.7 (4.3-49.0) | 89.3 (85.2-93.4) | 14.8 | 1.16 (0.5-2.6) | 81.0 | 26.8 |
| MAF5/PRO-C6 if MAF5 ≥1.0 | 50.9 | 15.2 | 11.4 (0.9-22.0) | 94.4 (91.1-97.6) | 26.7 (4.3-49.0) | 85.6 (80.9-90.3) | 11.4 | 0.89 (0.4-2.1) | 57.5 | 29.3 |
| NFS/PRO-C6 if NFS  ≥-1.455 | 55.3 | 16.4 | 10.8 (0.8-20.8) | 94.2 (90.8-97.5) | 26.7 (4.3-49.0) | 84.4 (79.5-89.3) | 10.8 | 0.84 (0.4-2.0) | 56.5 | 31.3 |
| Hospital care | | | | | | | | | | |
| FIB4/PRO-C6 if FIB4 ≥1.30 | 33.0 | 11.1 | 27.8 (13.1-42.4) | 89.9 (86.5-93.4) | 25.6 (11.9-39.3) | 90.9 (87.5-94.2) | 27.8 | 2.17 (1.0-4.5) | 32.4 | 10.7 |
| MAF5/PRO-C6 if MAF5 ≥1.0 | 70.4 | 22.6 | 26.4 (16.2-36.6) | 92.3 (88.9-95.6) | 50.0 (34.1-65.9) | 81.1 (76.5-85.7) | 26.4 | 2.06 (1.0-4.3) | 16.7 | 11.8 |
| NFS/PRO-C6 if NFS  ≥-1.455 | 62.0 | 18.1 | 27.6 (16.1-39.1) | 91.6 (88.3-95.0) | 42.1 (26.4-57.8) | 85.2 (81.0-89.3) | 27.6 | 2.16 (1.0-4.5) | 20.1 | 12.4 |

FIB4, fibrosis-4 index; MAF5, metabolic dysfunction–associated fibrosis-5 score; NFS, NAFLD-fibrosis score; PRO-C6, procollagen type VI N-terminal propeptide; PPV, positive predictive value; NPV, negative predictive value; NNS, number-needed-to-screen.

### **Supplemental table 26.** Diagnostic results and performance two-tiered care pathways using PRO-C6, stratified for the presence of T2DM.

|  | PRO-C6 needed, % | Referral to hepatology, % | PPV, % (95% CI) | NPV, % (95% CI) | Sensitivity, % (95% CI) | Specificity, % (95% CI) | Correct referral rate, % | Improved correct referral rate, RR (95% CI) | NNS for correct referral, whole care path, n | NNS for correct referral, after PRO-C6, n |
| --- | --- | --- | --- | --- | --- | --- | --- | --- | --- | --- |
| T2DM present | | | | | | | | | | |
| FIB4/PRO-C6 if FIB4 ≥1.30 | 36.2 | 10.8 | 31.0 (14.2-47.9) | 89.1 (85.2-93.1) | 25.7 (11.2-40.2) | 91.4 (87.8-95.0) | 31.0 | 0.99 (0.5-1.8) | 29.8 | 10.8 |
| MAF5/PRO-C6 if MAF5 ≥1.0 | 86.0 | 27.3 | 20.8 (11.5-30.2) | 90.1 (85.9-94.3) | 44.1 (27.4-60.8) | 75.2 (69.6-80.8) | 20.8 | 0.66 (0.3-1.3) | 17.6 | 15.1 |
| NFS/PRO-C6 if NFS  ≥-1.455 | 80.1 | 25.9 | 21.7 (12.0-31.5) | 89.8 (85.6-94.1) | 42.9 (26.5-59.3) | 76.6 (71.2-82.1) | 21.7 | 0.69 (0.4-1.3) | 17.7 | 14.2 |
| T2DM absent | | | | | | | | | | |
| FIB4/PRO-C6 if FIB4 ≥1.30 | 36.0 | 8.4 | 8.3 (0.0-19.4) | 89.5 (84.8-94.2) | 10.5 (0.0-24.3) | 86.8 (81.7-92.0) | 8.3 | 0.27 (0.1-0.6) | 143.0 | 51.5 |
| MAF5/PRO-C6 if MAF5 ≥1.0 | 40.1 | 8.1 | 17.4 (1.9-32.9) | 94.3 (91.4-97.1) | 21.1 (2.7-39.4) | 92.8 (89.7-95.9) | 17.4 | 0.55 (0.3-1.1) | 71.0 | 28.5 |
| NFS/PRO-C6 if NFS  ≥-1.455 | 39.5 | 8.2 | 8.7 (0.0-20.2) | 93.8 (90.9096.7) | 11.1 (0.0-25.6) | 92.0 (88.7-95.3) | 8.7 | 0.28 (0.1-0.6) | 140.5 | 55.5 |

FIB4, fibrosis-4 index; MAF5, metabolic dysfunction–associated fibrosis-5 score; NFS, NAFLD-fibrosis score; PRO-C6, procollagen type VI N-terminal propeptide; PPV, positive predictive value; NPV, negative predictive value; NNS, number-needed-to-screen; T2DM, type 2 diabetes mellitus.


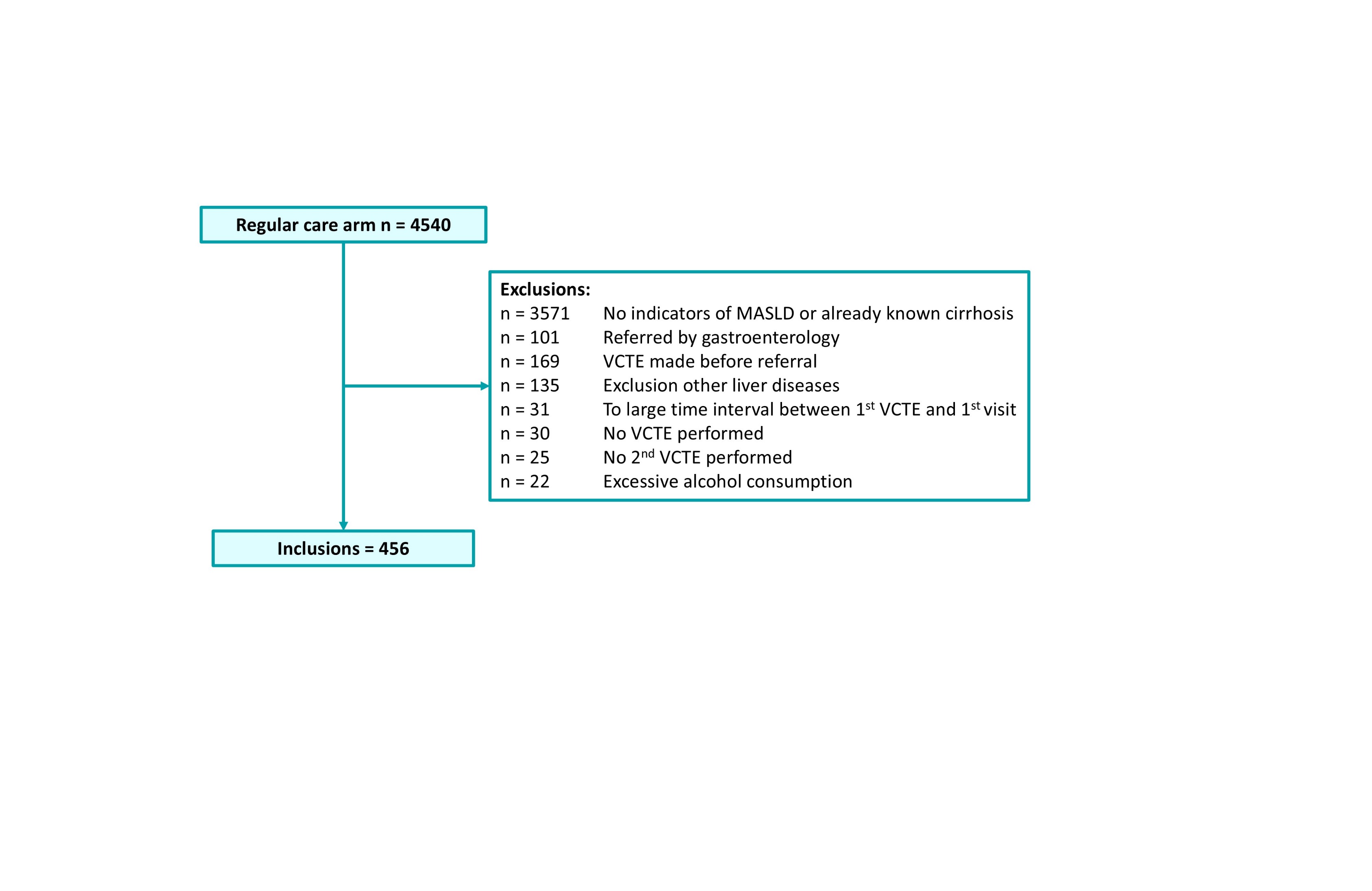


### **Supplemental figure 1.** Flow chart exclusion criteria regular care arm.

All referrals to the tertiary hepatology clinics, i.e. from primary, secondary, and tertiary care, were evaluated. Those referrals with a reason related to MASLD - specifically, referrals explicitly mentioning MASLD, those with an established diagnosis of steatosis, or those with liver function tests indicating steatosis (elevated AST and/or ALT) in the absence of other chronic liver diseases - were included in the comparison analysis. Patients were excluded when they had a previous diagnosis of ≥F3 fibrosis, when they were referred by a gastroenterologist, when a VCTE was performed prior to the referral, or when the first VCTE was performed ≥3 months after initial visit at the outpatient clinic. MASLD, metabolic dysfunction-associated steatotic liver disease; AST, aspartate transaminase; ALT, alanine transaminase; VCTE, vibration-controlled transient elastography.


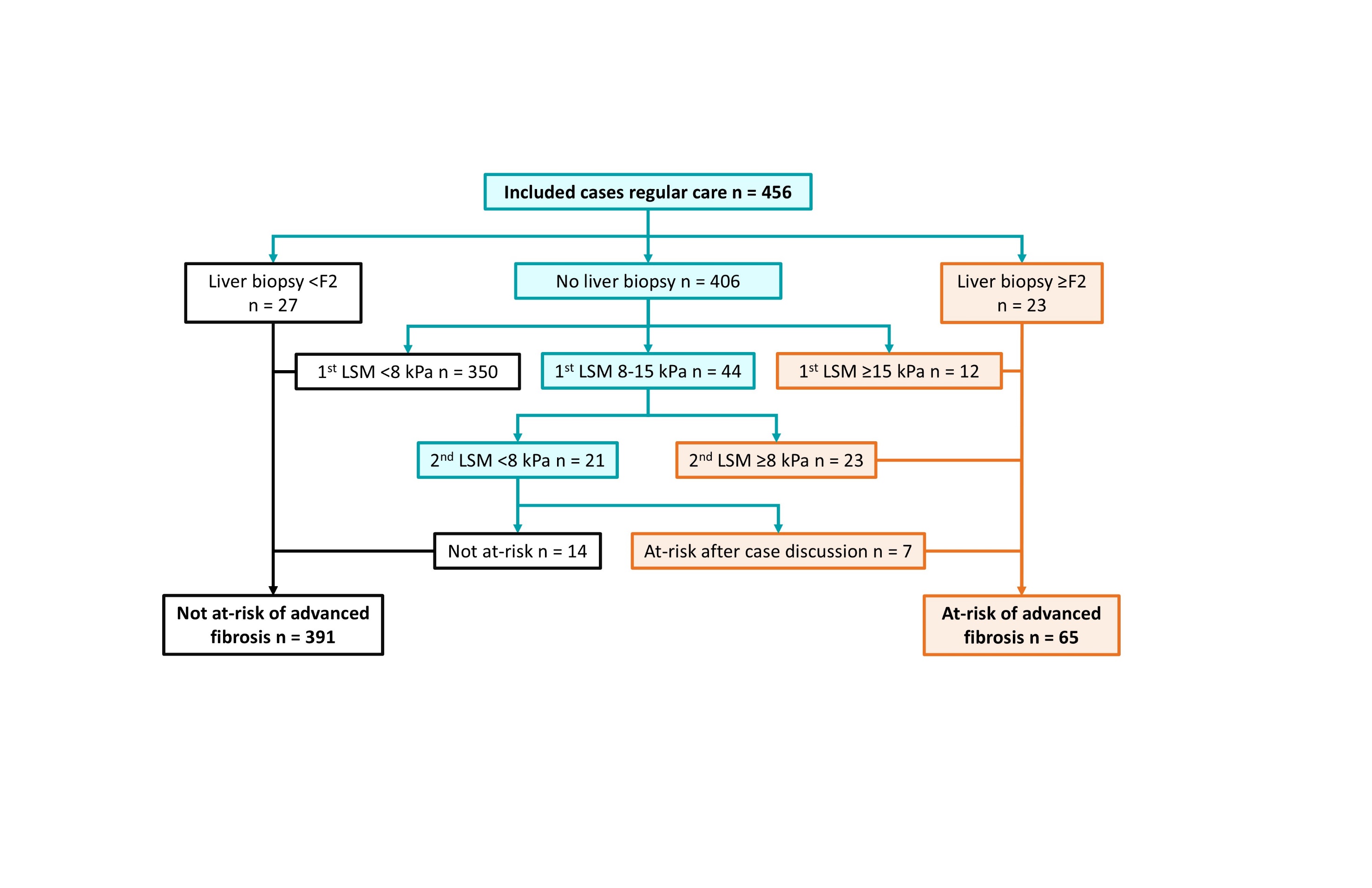


### **Supplemental figure 2.** Flow chart clinical reference standard regular care arm.

At-risk or at low-risk of advanced MASLD fibrosis was determined using a predefined composite hierarchical clinical reference standard comprising liver histology, imaging parameters and VCTE (LSM), depending on availability. MASLD, metabolic dysfunction-associated steatotic liver disease; LSM, liver stiffness measurement; VCTE, vibration-controlled transient elastography.
